# Self-perceived level of competitiveness, tension and dependency and depression risk in the SUN cohort

**DOI:** 10.1186/s12888-018-1804-x

**Published:** 2018-07-27

**Authors:** Francisca Lahortiga-Ramos, Cristian Raquel Unzueta, Itziar Zazpe, Susana Santiago, Patricio Molero, Almudena Sánchez-Villegas, Miguel Ángel Martínez-González

**Affiliations:** 1 Department of Nutrition, Food Science and Physiology, School of Pharmacy and Nutrition, University of Navarra, Pamplona, Navarra Spain; 2 Department of Preventive Medicine and Public Health, School of Medicine, University of Navarra, Pamplona, Navarra Spain; 3 Department of Psychiatry and Clinical Psychology, University Clinic of Navarra, Pamplona, Navarra Spain; 4 Navarra Institute for Health Research (IdisNA), Pamplona, Navarra Spain; 5 Nutrition Research Group, Research Institute of Biomedical and Health Sciences, University of Las Palmas de Gran Canaria, Las Palmas de Gran Canaria, Spain; 6 Biomedical Research Center Network on Obesity and Nutrition (CIBERobn) Physiopathology of Obesity and Nutrition, Institute of Health Carlos III, Madrid, Spain; 7 Department of Nutrition, Harvard School of Public Health, Boston, USA

**Keywords:** Depression risk, Competitiveness, Tension, Dependency

## Abstract

**Background:**

Emerging evidence suggests a possible etiologic role of certain personality traits (not necessary dysfunctional) in the risk of depression, but the longitudinal long-term available evidence is currently scarce. We longitudinally assessed whether 3 common personality traits (competitiveness, tension and dependency) were associated with the risk of depression after a maximum follow-up of 15 years.

**Methods:**

We assessed 15,604 university graduates free of depression at baseline through a self-administered questionnaire including personality traits. Simple, Likert-type, questions with 11 possible answers ranging from 0 to 10 were used at baseline to assess the 3 personality traits. We compared participants with high scores (7–10) versus those with low scores (0–4). New medical diagnoses of depression during follow-up were used as the outcome.

**Results:**

During a median follow-up of 10.1 y, we prospectively identified 902 new medical diagnoses of depression. The multivariable-adjusted hazard ratios (95% confidence intervals) for depression were 1.85 (1.52–2.24) for participants with higher baseline tension (7–10 versus 0 to 4), P-trend < 0.001; and 1.23 (1.06–1.44) for high versus low baseline dependence levels, P-trend = 0.004. Higher levels of competitiveness were marginally associated with lower risk of depression, with hazard ratio = 0.78 (0.61–1.01), P-trend = 0.105.

**Conclusion:**

A simple scoring system of personality traits shows an independent association with the future occurrence of depression. This finding underscores, with now prospective evidence, the importance of personality traits in the aetiology of depression and can provide a clinically useful tool for gathering valid information about depression-related personality traits.

**Electronic supplementary material:**

The online version of this article (10.1186/s12888-018-1804-x) contains supplementary material, which is available to authorized users.

## Background

Depression, is a major global public health problem and is characterized by lowered mood, loss of capacity to experience pleasure, increased sense of worthlessness, fatigue, and concerns about death and suicide [1]. Currently, the estimates are that 350 million people suffer depression [2], and depending on its level of intensity and duration it is a frequent and strong risk factor for suicide. Globally more than 800,000 persons commit suicide every year and suicide has become the first or second cause of mortality among young adults in most Western countries. In the Spanish adult population [3], the prevalence of chronic depression in 2014 was approximately 7.8% (4.8% of men and 10.7% of women). Taking these alarming data into account, depression represents a priority for public health and there is a need to broaden the etiologic research in the field of depression. One of the lines that nowadays is expanding, is the study of the relationship between depression and personality factors or traits (ways of thinking, feeling and behaving), which show heritability and stability across time [4–9]. Personality traits (especially competitiveness, psychological tension and dependency) are very likely to play a role in the aetiology and development of depression. The hypothesis that depression is linked to personality traits can be traced back to ancient times. Different models have been proposed to explain this association: one model hypothesizes that personality and depression share similar aetiology, but there is not a cause-effect relationship between them. A second model considers that personality does have a truly causal effect on depression onset and progression. Finally, a third model claims that personality and depression have a mutual “pathoplastic” relationship, meaning that each of them can influence the appearance of the other. Previous studies have shown associations between personality factors or specific personality traits and depression risk [10–15]. But these studies followed either cross-sectional designs or had only a short term follow-up. None of them used survival analyses in the long-term nor specifically appraised simultaneously the following 3 traits: dependency, tension and competitiveness in a well-defined cohort. Current evidence mainly suggests that depression is associated with higher levels of neuroticism and lower levels of extraversion, and to some extent, lower levels of agreeableness, measured according to the Five-Factor Model (FFM) [16]. The FFM evaluates five different dimensions of personality, also known as the Big Five personality domains: Neuroticism, Extraversion, Conscientiousness, Openness, and Agreeableness [11], but it does not include dependency, tension or competitiveness.

Only a few studies have aimed to ascertain associations of specific personality traits, such as competitiveness, tension and/or dependency/autonomy with the risk of depression [17–23]. As stated by Costa and McCrae, and also corroborated by Johnson [24], the assessment of the different facets explained in the Big Five Dimensions of the FFM, may lead to conclude that neuroticism includes anxiety, hostility, self-consciousness, impulsiveness, vulnerability, among others. Extraversion incorporates assertiveness, activity level, excitement-seeking, and positive emotions. And Conscientiousness includes competence, self-efficacy, order, dutifulness, achievement-striving, self-discipline, and cautiousness [16, 24]. Thus, it can be inferred that the 3 above-mentioned personality traits (competitiveness, tension, and dependency) represent a new field, though we acknowledged that they are also partly related to these FFM dimensions (conscientiousness, neuroticism and extraversion). Specifically, competitiveness trait is related to conscientiousness, tension trait would be associated with neuroticism, and dependency trait with the negative pole of conscientiousness and extraversion.

To our knowledge, previous studies have focused on analyzing the association between personality constructs or facets, such as extraversion, neuroticism or competitiveness, and depression but not in analyzing the relationship between these 3 specific personality traits (competitiveness, tension, and dependency) and the risk of developing depression in large longitudinal study. Our study aims at filling this existing gap and to investigate the independent association between levels of competitiveness, tension and dependence and the long-term risk of depression in a well-defined cohort of university graduates.

## Methods

### Participants

The Seguimiento Universidad de Navarra (SUN) project is a cohort study constituted by university alumni from different Spanish universities, and other professionals from different provinces in Spain. The recruitment of participants started in 1999 and is permanently open for new participants. Self-administered baseline (Questionnaire_0; Q_0) and follow-up questionnaires (Q_2-Q_16) are used to assess socio-demographic, diet and clinical characteristics of the subjects. The detailed methodology has been published elsewhere [25]. English version of SUN questionnaires are available in Additional file 1.

Up to December 2016, 22,564 participants had been recruited. Among them, 22,279 who had completed the Q_0 before March 1st of 2014 were considered for the present study (time frame needed for the participants to at least have one follow-up questionnaire completed). We excluded participants who were taking any antidepressant drug or were diagnosed with depression at baseline (*n* = 2621), and those without incident depression but were under antidepressant medication in any follow-up questionnaire (*n* = 285). Furthermore, all participants who reported an implausible energy intake (women < 500 kcal or > 3500 kcal, and men < 800 kcal or > 4200 kcal) (*n* = 1821), those with missing data in questions regarding personality traits (*n* = 357), and all subjects without at least one follow-up questionnaire completed (*n* = 1591) were also excluded. The final sample size available for statistical analyses was 15,604.

### Exposure assessment

Personality traits items were collected in Q_0 and included the following questions: a) Do you consider yourself a competitive, nonconformist, fighter person, who demands everything of yourself at work and sometimes even more of what you can afford?, b) Do you consider yourself a tense, aggressive, usually feeling overloaded, highly strung person or you think of yourself as a relaxed and calm person, and c) Do you think you have enough resources, preparation and autonomy to solve any problems at work, or do you exclusively depend on others to do it? For each question, 11 possible answers could be chosen by the participant ranging from 0 (more conformist, relaxed or autonomous) to 10 (more competitive, tense or dependent). For the present study, the concepts competitiveness, tension, and dependency will be used to describe the 3 personality traits assessed at baseline (Q_0). Finally, participants were categorized into 3 different groups according to their reported answers in the Q_0: low level (0–4), moderate level (5–6) and high level (7–10).

### Outcome assessment

Incidence of depression was assessed through the follow-up questionnaires (Q-2, Q-4, Q-6 and Q-14) with the following question: ‘Have you ever been diagnosed with depression by a medical doctor?’ All positive answers were considered new cases of depression. This question was previously validated in a subsample of the SUN cohort where 104 participants were also assessed by a Psychiatrist or a Clinical Psychologist through the Structured Clinical Interview for DSM-IV (SCID-I) [26].

The results showed that there were 46 true positives of the 62 self-reported cases of depression (74.2% (95% Confidence Interval (CI) 63.3, 85.1), and 34 true negatives of the 42 non-depressive participants (81.1% (95% CI = 69.1, 92.9).

### Covariate assessment

The Q_0 gathered information for many covariates, some of them potentially related to both personality traits and depression. These covariates could be potential confounding factors and we conducted multivariable-adjustments to control for them. Thus, the Q_0 also included a validated 136-item food frequency questionnaire (FFQ) to assess dietary habits. From these data, we calculated the adherence to the Mediterranean diet using the score proposed by Trichopoulou et al. [27]. A physical activity questionnaire (specifically validated in a subsample of the cohort) gathered information on frequency and type of activity. Furthermore, socio-demographic factors, anthropometric measurements, and personal and family clinical history, among other variables were also assessed at baseline. The validity of self-reported weight and body mass index (BMI) as well as the FFQ and physical activity questionnaire have been previously assessed [28–30].

### Statistical analysis

To describe baseline characteristics of participants, we used percentages for categorical variables, and mean and standard deviations for quantitative variables.

For each participant we computed person-years of follow-up from the date of returning the Q_0 to the date of depression diagnosis, the date of death or the date of returning the last follow-up questionnaire, whichever came first.

Cox regression models were used to assess the relationship between the 3 categories of each personality trait and the incidence of depression. Hazard ratios (HR) and their 95% CI were calculated for the scores of tension, dependency and competitiveness using the low level as the reference category. For the multiple-adjusted model, the following potential confounders were considered: Model 1 adjusted for age at recruitment (10 groups), sex, total energy intake (continuous variable), Model 2: additionally adjusted for BMI (continuous variable), tobacco consumption (never, former, current smoker < 15 cigarettes per day, and current smoker > 15 cigarettes per day), alcohol consumption (continuous variable), total physical activity per week (continuous variable), average time (hours/day) spent sitting down (continuous variable), Mediterranean diet score (continuous variable), total years of university education (continuous variable), whether the participant was a health professional (yes, no), marital status (single, married, and others), employment status (employed, unemployed/ retired, homemaker and student), baseline prevalence of diabetes (yes, no), prevalence of hypertension (yes, no), prevalence of hypercholesterolemia (yes, no), prevalence of cancer (yes, no). Model 3 additionally adjusted for fast food consumption (continuous variable), trans fatty acid intake (continuous variable), nutritional supplement intake (yes, no), snack consumption between main meals (yes, no), and following a special diet (yes, no). Model 4 additionally adjusted for the 2 other personality traits (low, moderate, high).

We conducted the following sensitivity analyses: 1) after excluding late cases of depression (> 10 y of follow-up), 2) after excluding cancer and cardiovascular disease cases at baseline, 3) subgroup analyses for men and women, 4) subgroup analyses for young and older participants (< 35 y or ≥ 35 y) and, 5) excluding early cases (2 first y).

Tests for linear trend across increasing categories of different personality traits were conducted by assigning the median punctuation within each category and treating this variable as continuous.

All tests were conducted with the statistical program STATA 12.1 and a *P*-value <0.05 was taken as statistically significant.

## Results

Baseline characteristics of participants according to their level of competitiveness, tension and dependency are described in Table 1.

**Table 1:** Baseline characteristics according to levels of competitiveness, tension and dependency on a sample of 15,604 participants from the SUN cohort [Mean (standard deviation) or percentage]

	Competitiveness			Tension			Dependency		
	Low (1–4)	Moderate (5–6)	High (7–10)	Low (1–4)	Moderate (5–6)	High (7–10)	Low (1–4)	Moderate (5–6)	High (7–10)
N	1170	3788	10,646	3852	4766	6986	10,407	1653	3544
Sex (% female)	63.3	63.8	57.1	55.9	62.3	58.9	54.1	68.0	58.4
Age (years)	37.3 (11.7)	37.5 (11.8)	37.2 (12.3)	36.5 (12.0)	37.0 (12.2)	37.9 (12.2)	37.4 (11.9)	34.3 (12.4)	38.2 (12.6)
BMI (kg/m^2^)	23.6 (3.5)	23.4 (3.5)	23.5 (3.5)	23.8 (3.5)	23.4 (3.4)	23.5 (3.5)	23.5 (3.4)	22.9 (3.2)	23.7 (3.6)
Marital status married (%)	52.6	52.1	50.2	48.9	48.6	53.5	51.8	39.0	53.5
Level of education (years)	4.9 (1.4)	4.9 (1.4)	5.1 (1.6)	5.0 (1.5)	5.0 (1.5)	5.1 (1.6)	5.1 (1.6)	4.8 (1.3)	5.0 (1.5)
Health professionals (%)	56.7	58.0	54.1	57.8	56.1	53.8	55.4	52.6	55.8
Employed (%)	78.2	77.9	78.9	77.7	77.7	79.7	79.9	69.8	78.8
Smoking status (%)									
Ex-smokers	23.2	26.2	24.3	21.0	24.4	26.7	24.8	19.5	26.6
Current smokers	26.1	26.1	23.5	25.0	16.6	15.9	15.9	17.5	16.9
Leisure-time physical activity (MET x h/week)	22.7 (19.1)	25.4 (21.5)	28.3 (25.0)	27.6 (24.8)	27.7 (24.4)	26.5 (22.9)	27.7 (24.3)	24.9 (21.9)	26.7 (23.3)
Sitting (h/day)	5.2 (2.0)	5.1 (2.0)	5.3 (2.1)	5.3 (2.0)	5.2 (2.1)	5.3 (2.1)	5.3 (2.0)	5.4 (2.1)	5.2 (2.0)
Prevalence of diabetes (%)	1.4	1.9	1.8	1.5	1.8	1.9	1.6	1.6	2.2
Prevalence of hypertension (%)	6.1	7.6	8.3	6.8	7.3	9.1	8.1	7.0	8.2
Prevalence of hypercholesterolemia (%)	16.0	16.0	16.7	14.4	15.2	18.4	16.5	14.2	17.4
Prevalence of cancer (%)	3.3	3.4	3.6	3.9	3.1	3.7	3.7	2.7	3.4
Weight gain ≥3 kg in last 5 years (%)	32.1	29.5	29.6	30.5	29.0	30.0	29.6	30.1	30.2
Mediterranean diet score	4.2 (1.8)	4.2 (1.7)	4.3 (1.7)	4.2 (1.7)	4.3 (1.7)	4.2 (1.7)	4.3 (1.7)	4.0 (1.7)	4.2 (1.7)
Dietary attitudes score	6.6 (2.6)	7.0 (2.5)	7.1 (2.5)	6.9 (2.5)	7.1 (2.5)	7.0 (2.5)	7.0 (2.5)	6.9 (2.5)	7.1 (2.5)
Following a special diet (%)	6.9	6.6	8.0	6.9	7.6	8.0	7.5	6.4	8.4
Nutritional supplements (%)	15.5	16.9	18.5	16.2	17.6	19.1	18.0	18.2	17.4
Snacking (%)	40.2	35.7	30.9	32.2	32.1	33.4	31.6	37.9	33.6
Dietary supplements (%)	15.5	16.9	18.5	16.2	17.6	19.1	18.0	18.2	17.4
Total energy intake (kcal/day)	2404 (609)	2335 (618)	2351 (614)	2365 (625)	2339 (608)	2352 (614)	2357 (613)	2369 (619)	2329 (619)
Protein (% E)	17.8 (3.2)	18.0 (3.2)	18.3 (3.3)	18.1 (3.4)	18.3 (3.2)	18.2 (3.3)	18.1 (3.2)	18.0 (3.3)	18.4 (3.3)
Carbohydrates (% E)	43.3 (7.3)	43.4 (7.3)	43.1 (7.3)	43.2 (7.3)	43.3 (7.2)	43.2 (7.4)	43.3 (7.3)	43.2 (7.5)	43.0 (7.3)
Fats (% E)	36.9 (6.7)	36.5 (6.5)	36.6 (6.5)	36.7 (6.5)	36.5 (6.4)	36.6 (6.5)	36.5 (6.4)	37.1 (6.9)	36.5 (6.4)
Cholesterol (mg/day)	422.2 (145.5)	408.9 (147.8)	417.5 (147.9)	417.5 (149.2)	412.5 (144.2)	416.9 (149.3)	414.9 (148.0)	419.9 (143.1)	416.3 (149.2)
Fiber (g/day)	27.5 (11.4)	27.5 (11.8)	27.9 (12.3)	27.6 (12.3)	28.1 (12.1)	27.7 (11.9)	27.9 (12.3)	27.1 (11.4)	27.6 (11.7)
Alcohol (g/day)	6.5 (10.4)	6.8 (10.3)	6.7 (9.9)	6.9 (9.9)	6.5 (9.6)	6.7 (10.4)	6.8 (10.0)	5.5 (9.6)	6.9 (10.4)

During 158,137 person-years of follow-up (mean follow-up: 10.1 y), we documented 902 incident cases of depression. The risks of incident depression during follow-up according to baseline levels of each personality trait are shown in Table 2.

**Table 2:** Hazard Ratios and Confidence Intervals (95%) of incident depression according to the level of competitiveness, tension and dependency

	Low	Moderate	High	P for trend
	(1–4)	(5–6)	(7–10)	
Competitiveness				
N	1170	3788	10,646	
Incidence of depression	72	217	613	
Person/years	12,075	38,901	107,161	
Crude rates	1 (ref)	0.90 (0.69–1.18)	0.92 (0.72–1.17)	0.747
Model 1	1 (ref)	0.90 (0.69–1.17)	0.95 (0.75–1.21)	0.826
Model 2	1 (ref)	0.89 (0.68–1.16)	0.96 (0.75–1.22)	0.721
Model 3	1 (ref)	0.89 (0.68–1.17)	0.95 (0.75–1.22)	0.782
Model 4	1 (ref)	0.84 (0.64–1.10)	0.78 (0.61–1.01)	0.105
Tension				
N	3852	4766	6986	
Incidence of depression	149	238	515	
Person/years	38,793	47,819	71,527	
Crude rates	1 (ref)	1.32 (1.08–1.63)	1.86 (1.55–2.23)	< 0.001
Model 1	1 (ref)	1.29 (1.05–1.58)	1.81 (1.51–2.18)	< 0.001
Model 2	1 (ref)	1.31 (1.07–1.61)	1.83 (1.53–2.20)	< 0.001
Model 3	1 (ref)	1.30 (1.06–1.60)	1.80 (1.50–2.17)	< 0.001
Model 4	1 (ref)	1.32 (1.07–1.62)	1.85 (1.52–2.24)	< 0.001
Dependency				
N	10,407	1653	3544	
Incidence of depression	547	117	238	
Person/years	105,498	17,049	35,592	
Crude rates	1 (ref)	1.36 (1.11–1.67)	1.30 (1.12–1.52)	< 0.001
Model 1	1 (ref)	1.31 (1.07–1.60)	1.30 (1.12–1.52)	< 0.001
Model 2	1 (ref)	1.31 (1.06–1.61)	1.30 (1.12–1.52)	< 0.001
Model 3	1 (ref)	1.30 (1.06–1.59)	1.30 (1.11–1.52)	< 0.001
Model 4	1 (ref)	1.24 (1.01–1.54)	1.23 (1.06–1.44)	0.004

Model 1: Adjusted for age at recruitment (10 groups), sex, total energy intake (continuous variable)

Model 2: Additionally adjusted for BMI (continuous variable), tobacco consumption (never, former, current smoker < 15 cigarettes per day, and current smoker > 15 cigarettes per day), alcohol consumption (continuous variable), total physical activity per week (continuous variable), sedentariness (continuous variable), Mediterranean diet score (continuous variable), total years of college (continuous variable), health professionals (yes, no), marital status (single, married, and others), employment status (employed, unemployed/ retired, homemaker and student), prevalence of diabetes (yes, no), prevalence of hypertension (yes, no), prevalence of hypercholesterolemia (yes, no), prevalence of cancer (yes, no)

Model 3: Additionally adjusted for fast food consumption (continuous variable), trans fatty acids intake (continuous variable), nutritional supplements intake (yes, no), snack consumption between main meals (yes, no), and follows a special diet (yes, no)

Model 4: Additionally adjusted for the other two personality traits according to assessed trait; competitiveness, (low, moderate, high), tension (low, moderate, high), o dependency (low, moderate, high)

No significant association was found between competitiveness and the incidence of depression, with very similar results both in the crude model and in the adjusted models. The adjusted HRs (95% CI) for moderate and high levels of competitiveness as compared to lower level were, respectively, 0.84 (0.64–1.10) and 0.78 (95% CI: 0.61–1.01) in model 4 (*P* for trend = 0.105).

On the other hand, participants in the moderate and in the high category of tension exhibited a significant direct association with the risk of depression during follow-up as compared to those in the lowest category (HR = 1.32; 95% CI:1.07–1.62 and HR = 1.85; 95% CI: 1.52–2.24, respectively), *P* for trend < 0.001 in the fully-adjusted model.

Finally, in multivariate model 4, when we assessed the risk of depression according to categories of dependency, participants in the moderate and high levels presented a 24 and 23% relatively higher risk of developing depression than those in the lowest category (HR = 1.24; 95% CI: 1.01–1.54 and HR = 1.23, respectively; 95% CI: 1.06–1.44). The estimates showed a statistically significant linear trend (*P* = 0.004).

The adjusted HRs for high level of competitiveness, tension and dependency for each standard deviation was 0.95 (0.88–1.02), 1.36 (1.26–1.47) and 1.08 (1.02–1.16) (data not shown).

Finally, we performed several sensitivity analyses. The main results did not change when we excluded late cases of depression (> 10 y of follow-up), after excluding cancer and cardiovascular disease cases at baseline, and when we separately analysed men or women. When we repeated the analyses among young or older participants (< 35 y or ≥ 35 y) the risk of depression was always higher in participants with higher levels of tension and dependency independently of the age group considered. When early cases were excluded, only tension level was associated with depression (Table 3).

**Table 3:** Sensitivity analyses. Hazard Ratios and Confidence Intervals (95%) of incident depression according to the level of competitiveness, tension and dependency

	N	Incident	Low	Moderate	High	P for trend
		depression	(1–4)	(5–6)	(7–10)	
Competitiveness						
Main analyses Model 4	15,604	902	1 (ref)	0.84 (0.64–1.10)	0.78 (0.61–1.01)	0.105
Excluding late cases (> 10 y of follow-up)	7319	310	1 (ref)	1.15 (0.72–1.85)	0.96 (0.61–1.53)	0.368
Only men	6367	266	1 (ref)	1.00 (0.58–1.72)	0.86 (0.52–1.43)	0.338
Only women*	9237	636	1 (ref)	0.78 (0.57–1.07)	0.78 (0.58–1.05)	0.311
Age < 35 y	7550	444	1 (ref)	0.65 (0.46–0.92)	0.61 (0.44–0.84)	0.028
Age ≥ 35 y**	8054	458	1 (ref)	1.22 (0.78–1.89)	1.09 (0.71–1.66)	0.714
Excluding cancer and cardiovascular disease prevalent	14,853	857	1 (ref)	0.84 (0.64–1.11)	0.79 (0.61–1.02)	0.111
Excluding early cases (2 first y)	15,354	652	1 (ref)	0.84 (0.62–1.15)	0.76 (0.56–1.02)	0.067
Tension						
Main analyses Model 4	15,604	902	1 (ref)	1.32 (1.07–1.62)	1.85 (1.52–2.24)	< 0.001
Excluding late cases (> 10 y of follow-up)	7319	310	1 (ref)	1.11 (0.79–1.57)	1.42 (1.03–1.96)	0.017
Only men	6367	266	1 (ref)	1.11 (0.75–1.65)	1.88 (1.32–2.66)	< 0.001
Only women^¥^	9237	636	1 (ref)	1.40 (1.10–1.80)	1.81 (1.43–2.28)	< 0.001
Age < 35 y	7550	444	1 (ref)	1.25 (0.95–1.66)	1.76 (1.36–2.29)	< 0.001
Age ≥ 35 y^¥¥^	8054	458	1 (ref)	1.47 (1.08–2.00)	2.00 (1.50–2.67)	< 0.001
Excluding cancer and cardiovascular disease prevalent	14,853	857	1 (ref)	1.33 (1.07–1.65)	1.87 (1.53–2.29)	< 0.001
Excluding early cases (2 first y)	15,354	652	1 (ref)	1.36 (1.06–1.73)	1.86 (1.49–2.33)	< 0.001
Dependency						
Main analyses Model 4	15,604	902	1 (ref)	1.24 (1.01–1.54)	1.23 (1.06–1.44)	0.004
Excluding late cases (> 10 y of follow-up)	7319	310	1 (ref)	1.29 (0.89–1.88)	1.42 (1.10–1.84)	0.005
Only men	6367	266	1 (ref)	1.53 (1.02–2.31)	1.32 (1.00–1.74)	0.026
Only women^ǂ^	9237	636	1 (ref)	1.15 (0.90–1.47)	1.22 (1.01–1.47)	0.031
Age < 35 y	7550	444	1 (ref)	1.16 (0.88–1.54)	1.06 (0.84–1.33)	0.503
Age ≥ 35 y^ǂǂ^	8054	458	1 (ref)	1.36 (0.99–1.87)	1.40 (1.13–1.72)	0.001
Excluding cancer and cardiovascular disease prevalent	14,853	857	1 (ref)	1.30 (1.03–1.59)	1.30 (1.11–1.52)	0.001
Excluding early cases (2 first y)	15,354	652	1 (ref)	1.23 (0.96–1.57)	1.18 (0.98–1.41)	0.052

Model 1: Adjusted for age at recruitment (10 groups), sex, total energy intake (continuous variable)

Model 2: Additionally adjusted for BMI (continuous variable), tobacco consumption (never, former, current smoker < 15 cigarettes per day, and current smoker > 15 cigarettes per day), alcohol consumption (continuous variable), total physical activity per week (continuous variable), sedentariness (continuous variable), Mediterranean diet score (continuous variable), total years of college (continuous variable), health professionals (yes, no), marital status (single, married, and others), employment status (employed, unemployed/ retired, homemaker and student), prevalence of diabetes (yes, no), prevalence of hypertension (yes, no), prevalence of hypercholesterolemia (yes, no), prevalence of cancer (yes, no)

Model 3: Additionally adjusted for fast food consumption (continuous variable), trans fatty acids intake (continuous variable), nutritional supplements intake (yes, no), snack consumption between main meals (yes, no), and follows a special diet (yes, no)

Model 4: Additionally adjusted for the other two personality traits according to assessed trait; competitiveness, (low, moderate, high), tension (low, moderate, high), o dependency (low, moderate, high)

HR estimated with Cox regression and CI of 95%

**Fig. 1:**
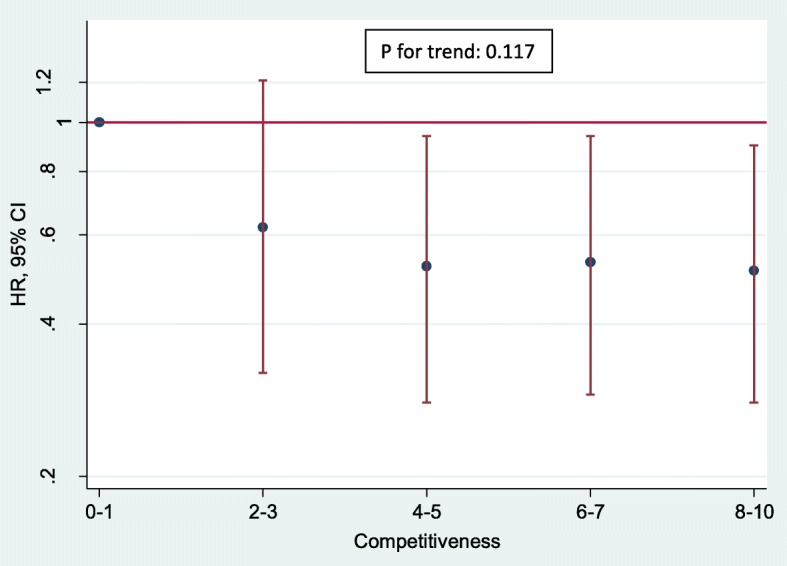
Hazard Ratios and Confidence Intervals (95%) of incident depression according to the level of competitiveness. Adjusted by age at recruitment (10 groups), sex, total energy intake (continuous variable), BMI (continuous variable), tobacco consumption (never, former, current smoker < 15 cigarettes per day, and current smoker > 15 cigarettes per day), alcohol consumption (continuous variable), total physical activity per week (continuous variable), sedentariness (continuous variable), Mediterranean diet score (continuous variable), total years of college (continuous variable), health professionals (yes, no), marital status (single, married, and others), employment status (employed, unemployed/ retired, homemaker and student), prevalence of diabetes (yes, no), prevalence of hypertension (yes, no), prevalence of hypercholesterolemia (yes, no), prevalence of cancer (yes, no), fast food consumption (continuous variable), trans fatty acids intake (continuous variable), nutritional supplements intake (yes, no), snack consumption between main meals (yes, no), follows a special diet (yes, no), level of tension (low, moderate, high) and level of dependency (low, moderate, high)

**Fig. 2:**
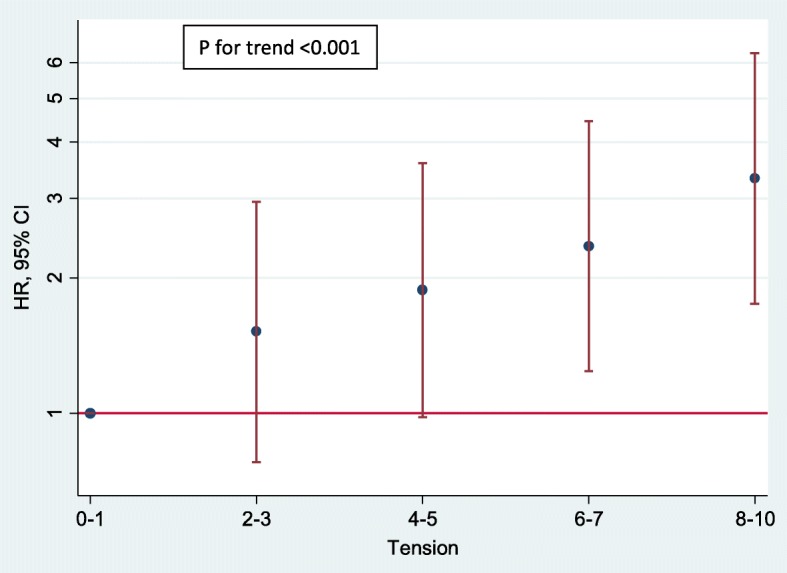
Hazard Ratios and Confidence Intervals (95%) of incident depression according to the level of tension. Adjusted by age at recruitment (10 groups), sex, total energy intake (continuous variable), BMI (continuous variable), tobacco consumption (never, former, current smoker < 15 cigarettes per day, and current smoker > 15 cigarettes per day), alcohol consumption (continuous variable), total physical activity per week (continuous variable), sedentariness (continuous variable), Mediterranean diet score (continuous variable), total years of college (continuous variable), health professionals (yes, no), marital status (single, married, and others), employment status (employed, unemployed/ retired, homemaker and student), prevalence of diabetes (yes, no), prevalence of hypertension (yes, no), prevalence of hypercholesterolemia (yes, no), prevalence of cancer (yes, no), fast food consumption (continuous variable), trans fatty acids intake (continuous variable), nutritional supplements intake (yes, no), snack consumption between main meals (yes, no), follows a special diet (yes, no), level of competitiveness (low, moderate, high) and level of dependency (low, moderate, high)

**Fig. 3:**
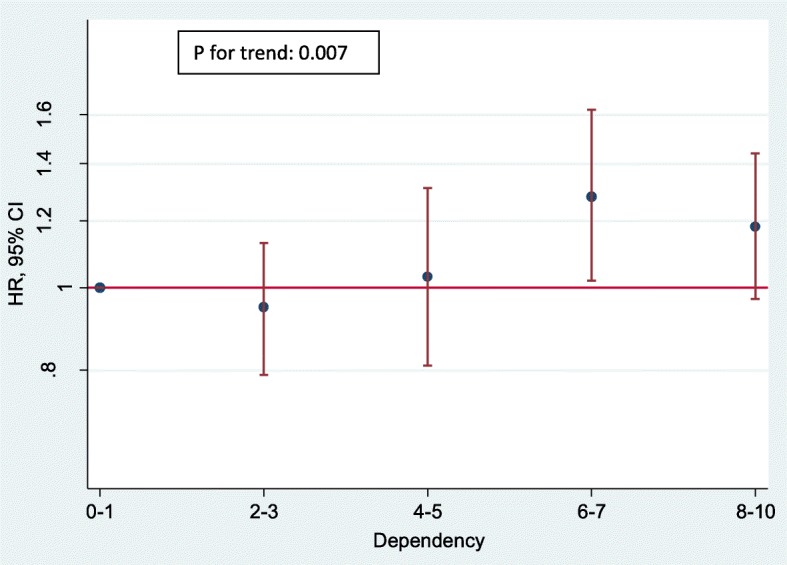
Hazard Ratios and Confidence Intervals (95%) of incident depression according to the level of dependency. Adjusted by age at recruitment (10 groups), sex, total energy intake (continuous variable), BMI (continuous variable), tobacco consumption (never, former, current smoker < 15 cigarettes per day, and current smoker > 15 cigarettes per day), alcohol consumption (continuous variable), total physical activity per week (continuous variable), sedentariness (continuous variable), Mediterranean diet score (continuous variable), total years of college (continuous variable), health professionals (yes, no), marital status (single, married, and others), employment status (employed, unemployed/ retired, homemaker and student), prevalence of diabetes (yes, no), prevalence of hypertension (yes, no), prevalence of hypercholesterolemia (yes, no), prevalence of cancer (yes, no), fast food consumption (continuous variable), trans fatty acids intake (continuous variable), nutritional supplements intake (yes, no), snack consumption between main meals (yes, no), follows a special diet (yes, no), level of competitiveness (low, moderate, high) and level of tension (low, moderate, high)

* p for interaction: 0.821; ** p for interaction: 0.087; ^¥^ p for interaction: 0.278; ^¥¥^p for interaction: 0.490; ^ǂ^ p for interaction: 0.363; ^ǂ ǂ^ p for interaction: 0.140;

When we assessed the association between each possible answer about the level of competitiveness and the incidence of depression, we found inverse associations after adjusting for several confounders with no significant linear trend (*P* for trend = 0.117) (Fig. 1). In these analyses, we also observed a direct association between a more tense personality (as compared to a more relaxed personality, with *P* for trend< 0.001) and between a more dependent personality ad compared to a more autonomous personality (*P* for trend = 0.007) (Figs. 2 and 3 respectively).

## Discussion

In this cohort composed of highly educated middle-aged adults, higher levels of self-perceived levels of dependency and tension were significantly associated with a higher risk of incident depression. These traits were assessed with short and simple Likert-type tools at baseline and showed long-term prospective associations with the future risk of depression, representing a novel finding.

However, our findings regarding the association of the personality traits and the risk of developing depression are in line with some previous investigations [15, 21, 22, 31, 32] that reported a higher incidence of depression in subjects who tend to have a more dependent trait.

Additionally, a greater level of psychological tension has been significantly associated with higher rates of depression [18]. Our results are also in line with other studies [14, 24] that have assessed the different facets of the FFM and that have found that higher levels of anxiety (Neuroticism facet), lower positive emotion (Extraversion facet), and lower conventionality (Conscientiousness facet) were prospective predictors of depressive illness.

Several novel methodological characteristics of our prospective study deserve to be underlined. These characteristics include the rigorous control of potential confounding by baseline variables using a comprehensive (Q_0), the elimination of prevalent cases of depression at baseline and the use of statistical methods of survival analyses (Cox regression models) recommended for prospective studies. The design and sensitivity analyses that we conducted support that the identified new cases of depression are in fact new cases of incident depression and mitigate the threat of reverse causality bias. As consequence, the specific personality traits that have been found to have a significant association, are characteristics of personality prior to the onset of the disorder and can be considered to be vulnerability factors that predispose to the future development of depression. While the hypothesis of depression and personality sharing a similar etiology cannot be discarded, our findings seem to be more in line with the model that considers personality as a causal factor for the future development of depression. This is what some authors have described as pre-depressive personality traits. In conjunction with stressful socio-environmental factors, these traits could lead to the development of the depressive symptoms [14, 15, 18].

However, the competitiveness trait only showed a marginal inverse association with depression, that was not statistically significant. This association was more apparent among participants <35 y, which can be explained by the existence of lower requirements at the family and professional level, typical of this age subgroup, but the p for interaction with age was not statistically significant (*p* = 0.087).

We consider that the assessment of these 3 personality traits through 3 short and simple Likert-type questions is a novel contribution that can be useful in many clinical settings. The results obtained in our study suggest that these specific personality traits, as assessed with these simple questions may represent key vulnerability factors to depression onset, and our assessment tool may be an efficient approach for collecting useful information on these traits in clinical evaluations.

Furthermore, the statistical analysis was carried out controlling for lifestyles (diet, physical activity, alcohol and tobacco consumption) and sociodemographic variables that could act as confounder variables for the association between personality and incidence of depression. To our knowledge, there are no other studies conducted with a similar methodology as ours.

We found that participants with moderate or high dependency levels had a greater risk of developing depression compared to the less dependent participants. However, the risk was lower in highly dependent subjects than in the moderately dependent participants. These results contrast with the finding by Eagleton et al. [33] who observed that higher autonomy (or less dependency) was associated with a higher incidence of depressive symptoms. Notwithstanding, another study conducted by Enns et al. [19] showed similar results to our findings and they reported that less autonomous persons, tend to show a greater incidence of depression compared to the individuals with a higher autonomy. Our results for dependency might be modified by the age of the participants (p for interaction = 0.140). Thus, in the subgroup analyses performed, we observed that among older subjects (> 35 y) the relative risk was significant only among older participants. This finding may suggest that it might be the maintenance of this trait in the long term, and the behaviors that derive from it, which increase the vulnerability for developing depression. The tension trait, as defined in our study, coincides largely with the neuroticism factor investigated by different authors, such as Eysenck [34] or Costa and Mcrae, in their model of the Big Five Factors [35] and which have frequently been associated with the presence of depression [36–38].

We acknowledge that the present study has several limitations. First, self-reporting of a clinical diagnosis was used as the criteria to establish depression, but it was previously validated and it is likely to have enough specificity given the high educational background of participants and the fact that more than 50 % of them are health professionals themselves [25]. Moreover, the fact that our participants are highly educated and highly motivated subjects makes unlikely that they may have misreported their correct diagnosis. We think that the optimal procedure to diagnose depression is in fact, the use of a proper general psychiatric interview made by a specialist in psychiatry or clinical psychology. However, the high number of participants in this study made this option less viable. To our knowledge scales that assess depressive symptoms such as the Diagnostic Inventory for Depression or Beck Depression Inventory are often used in most cohort studies as a diagnostic tool. An important limitation of these scales is that cut-off points to define depression status can be arbitrary, which also questions the validity of the diagnosis. Nonetheless, we consider our procedure to be a similar alternative option to assess the incidence of depression with such population size.

Second, the scores for self-perceived personality traits items were derived solely from the response given to three isolated items that report on these traits and not to the application of a standardized complete questionnaire, this may lead to some degree of measurement error. However, our results show as novel contribution that simple and short questions on key personality traits are in fact able to classify participants in categories associated with different risk for the future development of depression.

Dependency trait in our study refers specifically to the workplace, and we acknowledge that excluding other daily life environments is clearly a limitation. The participants that completed the Q_0 were in high proportion young adults, starting their professional career (3592 participants with a mean age inferior to 25 years). We assumed that during this age range, the self- assessment of dependency trait would be better identified in the workplace, instead of other environments more predominant later in life such as the family context. Enns et al. [19] have also assessed the dependency trait in the work environment.

Third, residual confounding is still possible in our study, but we attempted to adjust for the major depression risk factors and other potential confounders to provide utmost validity to our results. Finally, the homogeneity of participants in the SUN cohort (all university graduates) may limit the generalizability of our findings to the general population, although it also increases internal validity because the restriction to university graduates reduces many potential sources of confounding.

Despite these limitations, the strengths of this cohort study are the high retention rate, its prospective design and the large sample of participants highly educated who may be able to provide reliable self-reported data.

## Conclusions

In conclusion, self-perceived levels of dependency and tension as personality traits were significantly associated with an increased risk of depression and can be considered as vulnerability factors for future depressive illness development.

Future studies focused on these personality traits are needed to confirm a possible etiologic relationship between these traits and depression.

## Additional file

Additional file 1: SUN Questionnaires English version. (DOCX 73 kb)

## Abbreviations

CI: Confidence Intervals
HR: Hazard Ratios
Q_0: Baseline Questionnaire
WHO: World Health Organization
