# Supplementary material for: Self-perceived level of competitiveness, tension and dependency and depression risk in the SUN cohort
Source: BMC Psychiatry. 2018 Jul 27;18:241. doi: 10.1186/s12888-018-1804-x (PMC6062999; doi:10.1186/s12888-018-1804-x)
Supplement: Supplementary file 1 — SUN Questionnaires English version. (DOCX 73 kb) [file 12888_2018_1804_MOESM1_ESM.docx]

**SUN Questionnaires English**

- SUN Questionnaires English
- Baseline and follow-up questionnaires
- Self-administered baseline and follow-up questionnaires with socio-demographic, diet and clinical characteristics of the subjects

**First questionnaire. Project SUN (Seguimiento Universidad de Navarra)**

1. Sex
   1. Male
   2. Female
2. City of residence
   1. Zip code
   2. How many years have you lived in your city of residence?
3. Date of birth (day, month, year)
4. Weight (Kg)
5. Height (cm)
6. Have you had weight changes in the last five years?
   1. No change
   2. Lost weight: 1-2kg
   3. Lost weight: 3-4kg
   4. Lost weight: 5-10kg
   5. Lost weight: >10kg
   6. Gained weight: 1-2 kg
   7. Gained weight: 3-4kg
   8. Gained weight: 5-10kg
   9. Gained weight: >10kg
   10. Gained weight due to pregnancy
7. Civil status
   1. Single
   2. Married
   3. Widowed
   4. Divorced
   5. Other
8. Number of children
   1. None
   2. 1….9
   3. 10 or more
9. How many people live in your household including you?
   1. What is your highest level of education?
      1. I have not earned a diploma or licensure
      2. Doctorate
      3. Licensure/technical school
      4. Diploma (engineering-university colleges)
   2. Have you completed one of the following studies?
      1. Medicine
      2. Pharmacology
      3. Biology
      4. Nursing
      5. Nutrition
      6. Other biomedical studies
      7. No biomedical studies
10. What is your employment status?
    1. Full time
    2. Part time
    3. Homemaker
    4. Unemployed
    5. Retired
    6. Student
11. Have you smoke 100 cigarettes or more in your lifetime?
    1. No
    2. Yes, I continue to smoke
    3. Yes, I no longer smoke

Number of cigarettes/average number of cigarettes smoked each age

How long ago did you quit smoking?

Brand of cigarettes (for example: Marlboro Lights)

Please do not mark this shaded zone.

1. Do you smoke using a pipe or a cigar?
   1. Yes
   2. No
2. Have you regularly lived with a smoker? If yes, please indicate the number of years you have exposed to “second hand smoke”, and the approximate average number of hours that you have been exposed on a daily basis.
   1. At home: passive exposure

| Never been exposed | Years of exposure | Hours exposed daily |
| --- | --- | --- |
| Husband/wife smoker |  |  |
| Other smokers at home |  |  |

- 1. At work: passive exposure

| Never been exposed | Years of exposure | Hours exposed daily |
| --- | --- | --- |
| Only one colleague smoke in the same room |  |  |
| Several colleagues smoked in the same room |  |  |

1. On average in a typical week, how many days per week do you consume alcohol (wine, beer or liquor) including beverages with meals?
   1. Never, or almost never
2. How many days per week do you consume wine with meals?
3. What is the maximum number of alcoholic beverages (including wine, beer, and liquor) that you drink in one weekday?

In one weekend day?

In one special occasion (celebration, wedding, festivity)?

1. Do you drink alcoholic beverages and drive?
   1. I do not drive
   2. Almost never
   3. Yes, sometimes
   4. No, absolutely never
2. Do you utilize the following when travelling by car?
   1. Seat belt (yes, not always, almost never)
   2. Airbag (yes, no)
3. When you exercise or play sports, what do you think is your average level of intensity? Rate on a scale of 0 (minimum) to 10 (maximum).
   1. I never play sports or exercise
4. How long do you usually walk daily?
5. Your usual pace of walking is …
   1. Slow
   2. Normal, moderate
   3. Fast
   4. Very fast
6. How many stairs do you climb in total daily?
   1. 2 or less …
   2. 15 or more …
7. On average how many kilometer annualy do you dive or ride in a automobile?

On a motorcycle?

1. Total cholesterol level (mg/dL) (only labs performed less than 5 years ago)
   1. I have not had labs drawn
   2. I do not remember
   3. < 180 (low)
   4. 180-200 (normal)
   5. 200-240 (somewhat elevated)
   6. 241-300 (elevated)
   7. >300 (very elevated)

And HDL (mg/dL)

1. Pulse at rest (beats/minute, heart rate)
2. Current medications (mark only daily medications)
   1. I do not take any daily medications
   2. Aspirin > 2 times per week
   3. Other analgesics
   4. Cholesterol reducers
   5. Inulin
   6. Oral hypoglycemics (diabetic medications)
   7. Digoxin-Digitalis
   8. Diuretics
   9. Beta-Blockers (Metoprolol, Carvedilol…)
   10. Calcium channel blockers (amlodipine, diltiazem…)
   11. Nitrates (patches, sublingual, spray, tablets…)
   12. Other antithypertensives
   13. Weight loss medications
   14. Antidepressants
   15. Tranquilizers or sleep inducerds
   16. Other
   17. If you take other daily medications, please list the dose, frequency and duration on a separate sheet of paper.
3. Do you exercise?
4. On average, how much time have you dedicated to the following activities in the past year?
   1. Walk or stroll outdoors (including golf)
   2. Run or jog slowly
   3. Run competitively or quickly (athletics, etc.)
   4. Ride a bicycle
   5. Ride a stationary bicycle
   6. Swim
   7. Tennis, racquet ball, squash, other racket or ball sports
   8. Soccer or futsal
   9. Other team sports (basketball, handball…)
   10. Ballet, dancing, aerobics
   11. Hiking
   12. Gymnastics
   13. Yard work or pool maintenance, home improvement
   14. Skiing, ice skating
   15. Judo, karate or other marshal arts
   16. Sailing
   17. Other activities not listed
5. Average time dedicated to the following activities in the past year. Distinguish and answer both weekday and weekend.

| Time a day | A typical work day | A typical weekend day |
| --- | --- | --- |
| Watch TV-movies |  |  |
| Sitting at a computer |  |  |
| Driving |  |  |
| Sitting (in total) |  |  |
| Sleeping at night |  |  |
| Napping |  |  |
| Sun bathing (summer) |  |  |
| Sun bathing (winter) |  |  |
| Going out with friends |  |  |
| Standing at work |  |  |
| Domestic chores |  |  |
| Work activities more intense than standing |  |  |

1. Actual blood pressure (mmHg) (only if measured < 2 years ago)

Systolic (maximum)

- 1. I have never measured
  2. It was measured but I do not rember

Diastolic (minimum)

1. Have you undergone one of the following tests or preventative interventions without needing them for a specific illness? Mark each time that you underwent the intervention (age)
   1. General medical exam
   2. Electrocardiogram
   3. Stress test
   4. Chest x-ray
   5. Fecal occult blood test
   6. Colonoscopy/sigmoidoscopy
   7. Dental exam
   8. Intraocular pressure
   9. (only women) cervical cytology (pap smear)
   10. (only women) mammogram
   11. (only men) prostate exam
       1. Rectal exam
       2. Ultrasound
       3. Prostatic antigen
2. Has a medical professional diagnosed you with any of the following diseases?
   1. Diabetes
   2. Hypertension
   3. High cholesterol
   4. High triglycerides
   5. Myocardial infarction
   6. Angina pectoris
   7. Cardiopulmonary bypass surgery
   8. Coronary angioplasty
   9. Cerebral vascular accident (thrombosis-embolic-hemorrhagic)
   10. Paroxysmal tachycardia
   11. Atrial fibrillation
   12. Aortic aneurysm
   13. Heart failure
   14. Pulmonary embolism
   15. Deep vein thrombosis
   16. Intermittent claudication (peripheral vascular disease)
   17. Motor vehicle accident with fracture or hospitalization > 24hr
   18. Hip fracture
   19. Rheumatoid arthritis
   20. Colon or rectal polyps
   21. Gastric or duodenal ulcers
   22. Bronchial asthma
   23. Chronic bronchitis-emphysema
   24. Gall stones
   25. Kidney stones or renal colic
   26. Depression
   27. Cataracts
   28. Obesity
   29. Sleep apnea
   30. Cancer or tumor (indicate the type)
3. Have any of your relatives had the following disease?
   1. Myocardial infarction/sudden cardiac death (mother, father, sister, maternal grandmother, paternal grandmother)
   2. Breast cancer
   3. Hypertension
   4. Diabetes
   5. Melanoma
   6. Lung cancer
   7. Colorectal cancer
   8. Colorectal polyps
   9. Obesity
4. Which of the following diagrams best represents your body habitus at each age?
5. When you sunbathe, how often do you use sunscreen?
   1. I do not sunbathe
6. During childhood or adolescence, after sunbathing for >2hr without sunscreen, what type of skin reaction did you have?
   1. Basically none
   2. Only minor erythema
   3. Sun burn
   4. Severe, painful sun burn
   5. Severe, painful sun burn with blisters
7. Between ages 15-20, how many have you had severe sunburn with blisters?
8. Can you please count how many moles do you have from the knees to the ankles adding both legs?
   1. I feel uncomfortably counting them
   2. None
   3. 1….
9. Do you consider yourself a competitive, high achieving, determined person that commits one´s entire self to one´s work, including more than one can handle? Mark 0 (most satisfied) to 10 (most competitive).
10. Do you consider yourself a tense, aggressive person that excessively worries about things, or are you a person that is usually relaxed and calm? Mark 0 (most relaxed) to 10 (most tense)
11. Do you feel you have the necessary resources, preparation and autonomy to resolve the problems that present themselves at work, or do you exclusively depend on others? Mark 0 (most autonomous) to 10 (most dependent).
12. Excluding domestic chores, how many hours do you work weekly?
    1. Unemployed
13. How many days do you go home for lunch?
14. Do you suffer from or have you suffered from insomnia?
    1. Never
    2. Rarely
    3. Yes, and I continue to suffer
    4. Yes, in the past but no longer suffer
15. Do you snore at night?

Only women should answer questions 47 to 54 (lightly shaded zone).

1. Age of menarche (first period)
   1. If menopausal (cessation of periods), at what age did menopause occur?
      1. I continue to have periods
   2. What was the cause of menopause?
      1. Natural
      2. Gynecologic surgery, extraction: uterus and ovaries, uterus only, ovaries only
   3. Chemotherapy or radiation therapy
   4. Other

Please do not mark this dark shaded zone

1. Have you ever taken any hormonal therapies?
   1. Never
   2. Previously
   3. Currently

If yes, for how many years?

Please, specify the brand

1. Have you even been diagnostic with fibrocystic breast or other benign disease of the breast?
   1. Was it confirmed by biopsy?
2. Number of pregnancies
3. Multiple gestations, identical twins/fraternal twins
   1. Yes
   2. No

Age at which you conceived them

1. Age of the pregnancies: list if at each age you completed a pregnancy of 6 or more months, or in the other column, mark if the pregnancy was less than 6 months, including miscarriages or abortions.
2. On average, how long has breast feeding lasted for your children?

***Right upper corner: in this quadrant, please fill in the following boxes as previously completed with the identification number provided on the first page***.

Diet survey

Please, mark only one option for each food. *For each food, mark the box that indicates the average consumption during the past year. We try take into account variation between summer/winter. For example, if you eat ice cream 4 times a week during the three months of summer, the average usage would be once a week per year.*

1. Whole milk (one cup, 200ml)
2. Half milk (one cup, 200ml)
3. Skim milk (one cup, 200ml)
4. Condensed milk (1 tablespoon)
5. Whipped cream or creamer (1/2 cup)
6. Milk shakes (one cup, 200ml)
7. Yogurt (1, 125g)
8. Low fat yogurt (1, 125g)
9. Petit suisse (1, 100g)
10. Curd or cottage cheese (half a cup)
11. Cream cheese or cheese slices (1 portion)
12. Other cheeses: cured or semi-cured (Manchego, Swiss cheese, etc) (50g)
13. White or fresh cheese (Burgos, goat cheese) (50g)
14. Custard, flan, pudding (1 cup, 200ml)
15. Ice cream (one)

Please, mark only one option for each food. *One plate or portion of a 100-250g, except when otherwise indicated.*

1. Chicken eggs (one)
2. Chicken or turkey WITH skin
3. SKINLESS chicken or turkey
4. Calf or cow meat
5. Pork meat
6. Lamb meat
7. Rabbit or hare
8. Liver
9. Other organs: brain, heart, etc.
10. Serrano ham
11. Baked ham (one slice)
12. Sausages (chorizo, salchichón, mortadela) (50g)
13. Sausages (50g)
14. Paté, foie-gras (25g)
15. Blood sausage (50g)
16. Hamburger (one)
17. Sobressada (50g), meatball (three)
18. Bacon, pancetta (50g)
19. White fish: whiting, hake, sea bream, grouper, flounder (1 plate, piece or portion)
20. Blue fish: sardines, tuna, mackerel, salmon (1 plate, piece or portion)
21. Cod
22. Salted or smoked fish: herring, salmon
23. Oysters, clams, mussels, etc (six)
24. Shrimp, lobster, crayfish
25. Octopus, calamari, squid, cuttlefish

*One plate or portion of 250g, except when otherwise indicated.*

1. Chard, spinach
2. Cauliflower, Brussel sprouts, broccoli
3. Lettuce, escarole, endive
4. Row tomato (one, 150g)
5. Carrots, pumpkin
6. Green beans
7. Eggplant, squash, cucumber
8. Peppers
9. Asparagus
10. Gazpacho
11. Other greens (borage, thistle)
12. French fries, home maid, bag (1 portion, 150g)
13. Baked or steamed potatoes (1 portion, 150g)

Please, mark only one option for each food. *1 plate or portion.*

1. Orange, grapefruit (one), or mandarin (two)
2. Banana
3. Apple, pear
4. Strawberries (six, fruit salad)
5. Peach, apricot, nectarine
6. Cherries, plums (1 plate)
7. Figs
8. Watermelon (1 slice, 200-250g)
9. Melon (1 slice, 200-250g)
10. Canned fruits (2 cans)
11. Dates, dry figs, raisins (150g)
12. Almonds, peanuts, hazelnuts, walnuts (50g)
13. Olives (ten)
14. Avocado
15. Mangos, papaya
16. Kiwis

How many days a week you do it fruit as dessert?

Please, mark only one option for each food. *1 plate or portion of 60g dry weight.*

1. Lentils
2. Chickpeas
3. Beans (pinto, white, black)
4. Peas
5. White bread (3 slices, 60g)
6. Wheat bread (3 slices, 60g)
7. Cereals (30g)
8. White rice (60g)
9. Pasta, noodles, macaroni, spaghetti (60g)
10. Pizza (1 slice, 200g)

Please, mark only one option for each food. 1 tablespoon or individual portion to deep, or dress salads, total:

1. Butter
2. Margarine
3. Olive oil
4. Sunflower oil
5. Corn oil
6. Lard
7. Other:

How often do you consume: fried foods at home, fried food at restaurants

At home, when frying, I use: olive oil, sunflower oil, corn oil, butter, margarine, others

Brand of olive oil that you usually use at home

Please, mark only one option for each food

1. Maria cookies (4-6 cookies, 50g)
2. Cookies with chocolate (4-6 cookies, 50g)
3. Packaged muffins (1-2 muffins)
4. Donuts (one)
5. Roll, croissant or other packaged pastries (one, 50g)
6. Home maid pastries or dessert
7. Cakes (one, 50g)
8. Churros (one portion, 100g)
9. Chocolates and truffles (30g)
10. Turron (1/8 bar)
11. Tea cookies, ice cream, mazapan (1 portion, 90g)

Please, mark only one option for each food

1. Glass of red wine
2. Glass of other type of wine
3. Glass of wine with meals
4. Beer (1 pitcher, 330ml)
5. Liquors: whisky, gin, cognac, licorice (1 glass, 50ml)
6. Carbonated beverages with sugar: coca cola, fanta (1 bottle, 200ml)
7. As above, but low calorie, diet beverages (1 bottle, 200ml)
8. Natural orange juice (1 glass, 200ml)
9. Other natural fruit juices (1 glass, 200 ml)
10. Bottled or canned fruit or vegetable juice (200ml)
11. Decaffeinated coffee (1 cup, 50ml)
12. Regular coffee (1 cup, 50ml)
13. Tap water (1glass, 200ml)
14. Bottled water (1 glass, 200ml)

List which brand of water bottle you usually drink.

Please, mark only one option for each food

1. Croquetas, bunuelas, empanadas
2. Soups and creams
3. Tomato sauce, ketchup (1 teaspoon)
4. Mayonnaise (1 teaspoon)
5. Spice: tabasco, red pepper
6. Salt (one pinch)
7. Sugar (one teaspoon)
8. Splenda
9. Jams (1 teaspoon)
10. Honey
11. Other foods frequently consumed

How frequently do you eat out?

Have you consumed vitamins and/or minerals (including calcium) regularly over the past year?

If yes, please list the brand

Normally, what do you do with the fat of cooked meat?

1. I eat it
2. I remove it

Do you try to consume much fiber?

Do you try to consume much fruit?

Do you try to consume much vegetables?

Do you try to consume much fish?

Do you usually eat snacks in between meals?

Do you follow a special diet? If yes, indicate the type of diet.

Do you avoid the use of butter?

Do you try to reduce your consumption of fat?

Do you try to reduce your consumption of meat?

Do you restrict salt in your diet?

Do you add sugar to some beverages?

Do you try to reduce consumption of sweets?

Do you have an email address?

Do you have access to the internet?

Finally, it is essential that you fill the three addresses as follows with your first and last name. It is absolutely necessary to have your three addresses so that we can send you another questionnaire in 2 years. You may also list your telephone number and email, if you have one.

Note: it is very important that you complete these three address in capital letters.

**Second questionnaire. Project SUN (Seguimiento Universidad de Navarra)**

1. Are you a participant of project SUN? To which group do you belong? (You can choose multiple options)
   1. Graduate or alumni of the University of Navarra
   2. Employee of the University of Navarra
   3. Member of ACUNSA
   4. School of Nursing
   5. I received the questionnaire by other means (please specify)
2. In the last two years, have you undergone the following tests?
   1. Complete colonoscopy
   2. Sigmoidoscopy
   3. Lipid (cholesterol) panel
   4. Blood pressure measurement
   5. Electrocardiogram
   6. None of the above
3. Date of birth
4. Weight
5. Since the previous survey, have you changed one of the following habits?

|  | Have not changed | Have increased | Have decreased | I do not know |
| --- | --- | --- | --- | --- |
| Consumption of milk/dairy products |  |  |  |  |
| Consumption of fruits and/or vegetables |  |  |  |  |
| Consumption of meat and/or sausages |  |  |  |  |
| Consumption of fish |  |  |  |  |
| Consumption of salt |  |  |  |  |
| Consumption of alcohol |  |  |  |  |
| Consumption of butter |  |  |  |  |
| Consumption of olive oil |  |  |  |  |
| Consumption of pastries not made at home |  |  |  |  |
| Physical activity (adding both work and leisure time) |  |  |  |  |
| Computer use |  |  |  |  |

1. Since the first questionnaire, have you been diagnosed for the first time by a doctor with one of the following diseases, or have you suffered from one of the following circumstances?
   1. Motor vehicle accident with hospitalization of >24 hr
   2. Other motor vehicle accident without hospitalization
   3. Sports injury requiring medical therapy
   4. Other accident with injury, including fracture
   5. Hypertension: increase of > 8.5 diastolic or 13 systolic
   6. Osteoporosis
   7. Elevated total cholesterol (>240mg/dL)
   8. Myocardial infarction
   9. Angina pectoris
   10. Cardiopulmonary bypass surgery
   11. Atrial fibrillation
   12. Aortic aneurysm
   13. Heart failure
   14. Pulmonary embolism
   15. Deep vein thrombosis
   16. Diabetes
   17. Cerebral vascular accident (thrombosis-embolic-hemorrhagic)
   18. Intermittent claudication (peripheral vascular disease)
   19. Medical referral for fertility issues
   20. Cataracts
   21. Development of myopia or increase of > ½ diopters of myopia
   22. Glaucoma
   23. Macular degeneration
   24. Chronic bronchitis or emphysema
   25. Asthma
   26. Gastric or duodenal ulcer
   27. Renal colic
   28. Gallstones
   29. Anorexia nervosa or bulimia
   30. Anxiety
   31. Depression
   32. Colorectal polyps
   33. Tumor (specify the type)
   34. Other illness
2. How many km do you travel by car or motorcycle each year?
3. When travelling by vehicle, do you wear a seatbelt?
4. When travelling by motorcycle, do you wear a helmet?
5. When travelling by bicycle, do you wear a helmet?
6. What medications or dietary supplements (including vitamins, fibers, wheat bran, etc) do you consume on a regular basis?
   1. I do not frequently consume medications, supplements or wheat bran
   2. I consume the following medications or supplements daily (if you need more space, please add a separate sheet)
7. Have you been seen by a doctor in the last 2 years?
   1. No
   2. Yes, at the Primary Care Clinic
   3. Yes, at the University of Navarra hospital
   4. Yes, another hospital/another doctor

Thank you very much for your valuable collaboration.

**Third questionnaire. Project SUN (Seguimiento Universidad de Navarra)**

1. In general, would you say your state of health is
   1. Excellent
   2. Very good
   3. Good
   4. Fair
   5. Poor
2. How would you say your current state of health is compared to 2 years prior:
   1. Much better now
   2. Somewhat better now
   3. More or less the same
   4. Somewhat worse now
   5. Much worse now
3. The following questions refer to activities or things that you participate in on a normal day. My health…

|  | Yes, it limits me very much | Yes, it limits me very little | No, it does not limit me |
| --- | --- | --- | --- |
| Limits intense activities like running, lifting heavy objects or participating in strenuous sports |  |  |  |
| Limits moderate activities like moving a table, vacuuming, bowling, or walking > 1hr |  |  |  |
| Limits my carrying of grocery bags |  |  |  |
| Limits my ability to climb multiple flights of stairs |  |  |  |
| Limits my ability to climb one flight of stairs |  |  |  |
| Limits my ability to bend over or kneel |  |  |  |
| Limits my ability to walk one km or more |  |  |  |
| Limits my ability to walk multiple blocks |  |  |  |
| Limits my ability to walk one block |  |  |  |
| Limits my ability to bathe or dress myself |  |  |  |

1. In the last 4 weeks…
   1. Have you had to reduce the time dedicate to work or to your daily activities due to your physical health?
   2. Have you had to do less than what you wanted to due to your physical health?
   3. Have you had to stop performing certain work duties or daily activities due to your physical health?
   4. Have you had difficulty performing work or daily activities (for example, did it take more effort than usual) due to your physical health?
   5. Have you had to reduce the time dedicated to work or your daily activities due to an emotional problem (for example, due to sadness, depression, or anxiety)
   6. Did you do less than you wanted to accomplish due to an emotional problem (for example, due to sadness, depression, or anxiety)
   7. Were you unable to accomplish work or daily activities as carefully as normal due to an emotional problem (for example, due to sadness, depression, or anxiety)
   8. To what extend have your physical health or emotional problems limited your social life with family, friends, neighbors, or other people?
      1. None
      2. Some
      3. Regularly
      4. Much
      5. Very much
2. Have you had pain in some part of your body in the last 4 weeks?
   1. No, none
   2. Yes, very little
   3. Yes, a little
   4. Yes, moderate
   5. Yes, much
   6. Yes, very much
3. To what extend has your pain limited your daily work (including work away from home or domestic chores)?
   1. None
   2. A little
   3. Regularly
   4. Much
   5. Very much
4. In the last 4 weeks…

|  | Always | Almost always | Often | Once in a while | Only once | Never |
| --- | --- | --- | --- | --- | --- | --- |
| How often did you feel lively? |  |  |  |  |  |  |
| How often did you feel anxious? |  |  |  |  |  |  |
| How often did you feel with such low morale that nothing could motivate you? |  |  |  |  |  |  |
| How often did you feel very energetic? |  |  |  |  |  |  |
| How often did you feel down and sad? |  |  |  |  |  |  |
| How often did you feel fatigued? |  |  |  |  |  |  |
| How often did you feel happy? |  |  |  |  |  |  |
| How often did you feel tired? |  |  |  |  |  |  |

1. In the last 4 weeks, have your physical health or emotional problems limited your social activities?
   1. Always
   2. Almost always
   3. Once in a while
   4. Only once
   5. Never
2. Please, state if you feel the following statements are true or false:

|  | Definitely certain | Very certain | I do not know | Very false | Totally false |
| --- | --- | --- | --- | --- | --- |
| I think I become sick easier than other people |  |  |  |  |  |
| I am as healthy as everyone else |  |  |  |  |  |
| I think my health will worsen |  |  |  |  |  |
| My health is excellent |  |  |  |  |  |

1. Since the previous questionnaire (see the date on the previous page), have you changed one of the following habits?

|  | Have not changed | Have increased | Have decreased | I do not know |
| --- | --- | --- | --- | --- |
| Consumption of milk/dairy products |  |  |  |  |
| Consumption of fruits and/or vegetables |  |  |  |  |
| Consumption of meat and/or sausages |  |  |  |  |
| Consumption of fish |  |  |  |  |
| Consumption of salt |  |  |  |  |
| Consumption of alcohol |  |  |  |  |
| Consumption of butter |  |  |  |  |
| Consumption of olive oil |  |  |  |  |
| Consumption of pastries not made at home |  |  |  |  |
| Physical activity (adding both work and leisure time) |  |  |  |  |
| Computer use |  |  |  |  |

1. Have you smoked a cigarette in the last 4 weeks?
   1. No, I have never smoked
   2. No, I stopped smoking since….
   3. Yes, I have smoked for…
      1. Number of cigarettes a day

On a regular basis…

1. How many km do you travel by car or motorcycle each year?
2. When travelling by vehicle, do you wear a seatbelt?
3. When travelling by motorcycle, do you wear a helmet?
4. When travelling by bicycle, do you wear a helmet?
5. Since the second questionnaire (see date on previous date) have you undergone the following tests?
   1. Complete colonoscopy
   2. Sigmoidoscopy
   3. Lipid (cholesterol) panel
   4. Blood pressure measurement
   5. Electrocardiogram
   6. None of the above
6. Have you been seen by a doctor in the last 2 years?
   1. No
   2. Yes, at the Primary Care Clinic
   3. Yes, at the University of Navarra hospital
   4. Yes, another hospital/another doctor
7. Since the second questionnaire (see date on the previous date), have you been diagnosed for the first time by a doctor with one of the following diseases, or have you suffered from one of the following circumstances?
   1. Motor vehicle accident with hospitalization of >24 hr
   2. Other motor vehicle accident without hospitalization
   3. Sports injury requiring medical therapy
   4. Other accident with injury, including fracture
   5. Hypertension: increase of > 8.5 diastolic or 13 systolic
   6. Osteoporosis
   7. Elevated total cholesterol (>240mg/dL)
   8. Myocardial infarction
   9. Angina pectoris
   10. Cardiopulmonary bypass surgery
   11. Atrial fibrillation
   12. Aortic aneurysm
   13. Heart failure
   14. Pulmonary embolism
   15. Deep vein thrombosis
   16. Diabetes
   17. Cerebral vascular accident (thrombosis-embolic-hemorrhagic)
   18. Intermittent claudication (peripheral vascular disease)
   19. Medical referral for fertility issues
   20. Cataracts
   21. Development of myopia or increase of > ½ diopters of myopia
   22. Glaucoma
   23. Macular degeneration
   24. Chronic bronchitis or emphysema
   25. Asthma
   26. Gastric or duodenal ulcer
   27. Renal colic
   28. Gallstones
   29. Anorexia nervosa or bulimia
   30. Anxiety
   31. Depression
   32. Colorectal polyps
   33. Tumor (specify the type)
   34. Other illness
8. What medications or dietary supplements (including vitamins, fibers, wheat bran, etc) do you consume on a regular basis?
   1. I do not frequently consume medications, supplements or wheat bran
   2. I consume the following medications or supplements daily (if you need more space, please add a separate sheet)
9. Have you been seen by a doctor in the last 2 years?
   1. No
   2. Yes, at a General or Family Medicine Clinic
   3. Yes, at a hospital or with a medical specialist
10. Since the previous questionnaire, have you been diagnosed for the first time by a physician with one of the following conditions or have you undergone one of the following circumstances (remember that the date of your previous questionnaire that you sent to us is attached to your identification number in the upper right portion of the first page. Thus, the date that you list regarding the following questions, should be after the aforementioned date).
    1. Motor vehicle accident with hospitalization of >24 hr
    2. Other motor vehicle accident without hospitalization but requiring leave of absence from work
    3. Sports injury requiring medical therapy
    4. Other accident with injury, including fracture
    5. Hypertension: increase of > 8.5 diastolic or 13 systolic
    6. Osteoporosis
    7. Elevated total cholesterol (>240mg/dL)
    8. Myocardial infarction
    9. Angina pectoris
    10. Cardiopulmonary bypass surgery
    11. Atrial fibrillation
    12. Aortic aneurysm
    13. Heart failure
    14. Pulmonary embolism
    15. Deep vein thrombosis
    16. Diabetes (excluding gestational diabetes)
    17. Gestational diabetes
    18. Cerebral vascular accident (thrombosis-embolic-hemorrhagic)
    19. Intermittent claudication (peripheral vascular disease)
    20. Pregnancy (list the date of delivery or the expected due date)
    21. Consultation for fertility issues
    22. Cataract surgery
    23. Diagnosis of cataracts without surgery
    24. Increase of > ½ diopters of myopia
    25. Glaucoma
    26. Macular degeneration
    27. Chronic bronchitis or emphysema
    28. Asthma
    29. Gastric or duodenal ulcer
    30. Renal colic
    31. Gallstones
    32. Anorexia nervosa or bulimia
    33. Anxiety
    34. Depression
    35. Colorectal polyps
    36. Malignant tumor (specify the type)
    37. Other illness (please specify)
11. What medications or dietary supplements (including vitamins, fibers, wheat bran, etc) do you consume on a regular basis?
    1. I do not frequently consume medications, supplements or wheat bran
    2. I consume the following medications or supplements daily (if you need more space, please add a separate sheet)
12. Please, indicate the following measurements that have been performed in the last two years:
    1. Waist circumference
    2. Hip circumference
    3. Blood pressure
    4. Basal blood glucose
    5. Cholesterol

**Fourth questionnaire. Project SUN (Seguimiento Universidad de Navarra)**

1. In general, would you say your state of health is
   1. Excellent
   2. Very good
   3. Good
   4. Fair
   5. Poor
2. How would you say your current state of health is compared to 2 years prior:
   1. Much better now
   2. Somewhat better now
   3. More or less the same
   4. Somewhat worse now
   5. Much worse now
3. Please mark how often you consume each food or group of foods:
   1. Butter, margarine, or cream (1 serving, 12g)
   2. Olive oil to cook, dress salads or add to bread (1 serving, 1 tablespoon)
   3. Homemade tomato sauce or stir-fry (garlic, onion, leek) prepared with olive oil
   4. Vegetables (1 serving, 200g)
   5. Legumes (lentils, chickpeas, beans, etc) (1 serving, 60g dry weight)
   6. Fruits
   7. Nuts (1 serving, 30g)
   8. Red meat, sausages (1 serving, 100-150g)
   9. Chicken, turkey or rabbit
   10. Seafood or fish
   11. Packaged pastries including cookies, cakes, flan, and sweet desserts
   12. Carbonated and/or sweetened beverages (coolers, sodas, tonics, bitters) excluding diet beverages (1 can, 33cl)
   13. Wine
   14. Beer
   15. Distilled liquors: whisky, gin, cognac, vodka
   16. How often do you eat out?
   17. How often are the above fast food restaurants (pizzerias, burger joints)?
4. With which do you prefer to cook? (only choose one option)
   1. Olive oil
   2. Seed oil (sunflower, soy, etc)
   3. Butter
   4. Margarine
   5. Other fats or oils
5. Have you smoked a cigarette in the last 4 weeks?
   1. No, I have never smoked
   2. No, I stopped smoking since….
   3. Yes, I have smoked for…
      1. Number of cigarettes a day
6. How many km do you travel by car or motorcycle each year?
7. When travelling by vehicle, do you wear a seatbelt?
8. When travelling by motorcycle, do you wear a helmet?
9. When travelling by bicycle, do you wear a helmet?
10. Since the third questionnaire (see date on the upper part of the first page) have you undergone the following tests?
    1. Complete colonoscopy
    2. Sigmoidoscopy
    3. Lipid (cholesterol) panel
    4. Blood pressure measurement
    5. Electrocardiogram

**Fifth questionnaire. Project SUN (Seguimiento Universidad de Navarra)**

1. How many children you have?
2. If you have myopia, hyper myopia or astigmatism, please indicate the number of diopters you have
3. In the last 2 years, have you had lost weight intentionally?
   1. No, I have not changed
   2. I have lost 1-2 kg
   3. I have lost 3-4 kg
   4. I have lost 5-10 kg
   5. I have lost more than 10 kg
4. In the last 2 years, have you lost weight unintentionally? (for example, due to illness, stress or depression)
   1. No, I have not changed
   2. I have lost 1-2 kg
   3. I have lost 3-4 kg
   4. I have lost 5-10 kg
   5. I have lost more than 10 kg

How often you eat out?

How often are the above fast food restaurants (pizzerias, burger joints)?

1. On average, how much time do you spend on personal work (read, study, etc) on a regular week day?
2. On average, how much time do you spend on personal work (read, study, etc) on a regular weekend day?
3. Have you smoked a cigarette in the last 4 weeks?
   1. No, I have never smoked
   2. No, I stopped smoking since….
   3. Yes, I have smoked for…
      1. Number of cigarettes a day
4. How many km do you travel by car or motorcycle each year?
5. When travelling by vehicle, do you wear a seatbelt?
6. When travelling by motorcycle, do you wear a helmet?
7. When travelling by bicycle, do you wear a helmet?
8. Since the previous questionnaire, have you been diagnosed for the first time by a physician with one of the following conditions or have you undergone one of the following circumstances (remember that the date of your previous questionnaire that you sent to us is attached to your identification number in the upper right portion of the first page. Thus, the date that you list regarding the following questions, should be after the aforementioned date).
   1. Motor vehicle accident with hospitalization of >24 hr
   2. Other motor vehicle accident without hospitalization but requiring leave of absence from work
   3. Sports injury requiring medical therapy
   4. Other accident with injury, including fracture
   5. Hypertension: increase of > 8.5 diastolic or 13 systolic
   6. Osteoporosis
   7. Elevated total cholesterol (>240mg/dL)
   8. Myocardial infarction
   9. Angina pectoris
   10. Cardiopulmonary bypass surgery
   11. Atrial fibrillation
   12. Aortic aneurysm
   13. Heart failure
   14. Deep vein thrombosis
   15. Pulmonary embolism
   16. Diabetes (excluding gestational diabetes)
   17. Gestational diabetes
   18. Cerebral vascular accident (thrombosis-embolic-hemorrhagic)
   19. Intermittent claudication (peripheral vascular disease)
   20. Pregnancy (list the date of delivery or the expected due date)
   21. Consultation for fertility issues
   22. Cataract surgery
   23. Diagnosis of cataracts without surgery
   24. Increase of > ½ diopters of myopia
   25. Glaucoma
   26. Macular degeneration
   27. Chronic bronchitis or emphysema
   28. Asthma
   29. Gastric or duodenal ulcer
   30. Renal colic
   31. Gallstones
   32. Anorexia nervosa or bulimia
   33. Anxiety
   34. Depression
   35. Colorectal polyps
   36. Malignant tumor (specify the type)
   37. Other illness (please specify)
9. In general, would you say your state of health is
   1. Excellent
   2. Very good
   3. Good
   4. Fair
   5. Poor
10. How would you say your current state of health is compared to 2 years prior:
    1. Much better now
    2. Somewhat better now
    3. More or less the same
    4. Somewhat worse now
    5. Much worse now
11. The following questions refer to activities or things that you participate in on a normal day. My health…

|  | Yes, it limits me very much | Yes, it limits me very little | No, it does not limit me |
| --- | --- | --- | --- |
| Limits intense activities like running, lifting heavy objects or participating in strenuous sports |  |  |  |
| Limits moderate activities like moving a table, vacuuming, bowling, or walking > 1hr |  |  |  |
| Limits my carrying of grocery bags |  |  |  |
| Limits my ability to climb multiple flights of stairs |  |  |  |
| Limits my ability to climb one flight of stairs |  |  |  |
| Limits my ability to bend over or kneel |  |  |  |
| Limits my ability to walk one km or more |  |  |  |
| Limits my ability to walk multiple blocks |  |  |  |
| Limits my ability to walk one block |  |  |  |
| Limits my ability to bathe or dress myself |  |  |  |

1. In the last 4 weeks…
   1. Have you had to reduce the time dedicate to work or to your daily activities due to your physical health?
   2. Have you had to do less than what you wanted to due to your physical health?
   3. Have you had to stop performing certain work duties or daily activities due to your physical health?
   4. Have you had difficulty performing work or daily activities (for example, did it take more effort than usual) due to you physical health?
   5. Have you had to reduce the time dedicated to work or your daily activities due to an emotional problem (for example, due to sadness, depression, or anxiety)
   6. Did you do less than you wanted to accomplish due to an emotional problem (for example, due to sadness, depression, or anxiety)
   7. Were you unable to accomplish work or daily activities as carefully as normal due to an emotional problem (for example, due to sadness, depression, or anxiety)
   8. To what extend do your physical health or emotional problems have limited your social life with family, friends, neighbors, or other people?
      1. None
      2. Some
      3. Regularly
      4. Much
      5. Very much
2. Have you had pain in some part of your body in the last 4 weeks?
   1. No, none
   2. Yes, very little
   3. Yes, a little
   4. Yes, moderate
   5. Yes, much
   6. Yes, very much
3. To what extend has your pain limited your daily work (including work away from home or domestic chores)?
   1. None
   2. A little
   3. Regularly
   4. Much
   5. Very much
4. In the last 4 weeks…

|  | Always | Almost always | Often | Once in a while | Only once | Never |
| --- | --- | --- | --- | --- | --- | --- |
| How often did you feel lively? |  |  |  |  |  |  |
| How often did you feel anxious? |  |  |  |  |  |  |
| How often did you feel with such low morale that nothing could motivate you? |  |  |  |  |  |  |
| How often did you feel calm and peaceful |  |  |  |  |  |  |
| How often did you feel very energetic? |  |  |  |  |  |  |
| How often did you feel down and sad? |  |  |  |  |  |  |
| How often did you feel fatigued? |  |  |  |  |  |  |
| How often did you feel happy? |  |  |  |  |  |  |
| How often did you feel tired? |  |  |  |  |  |  |

1. Please, state if you feel the following statements are true or false:

|  | Definitely certain | Very certain | I do not know | Very false | Totally false |
| --- | --- | --- | --- | --- | --- |
| I think I become sick easier than other people |  |  |  |  |  |
| I consider myself as a person of will power |  |  |  |  |  |
| I am as healthy as everyone else |  |  |  |  |  |
| I think my health will worsen |  |  |  |  |  |
| My health is excellent |  |  |  |  |  |

1. In the last 4 weeks, how often have your physical health or emotional problems limited your social life (for example, to visit relatives or friends)?
   - 1. Always
     2. Almost always
     3. Sometimes
     4. Few time
     5. Never
2. Please, indicate the following measurements that have been performed in the last two years:
   1. Waist circumference
   2. Blood pressure
   3. Basal blood glucose
   4. Lipid panel: total cholesterol, LDL, HDL, triglycerides

C_10 questionnaire. Project SUN

Instructions:

Fill in the boxes ONLY with pencil.

Completely erase incorrect answers.

Leave in blank the box on the right upper quadrant of this sheet.

Do not fold this sheet.

Fill in the boxes correctly.

Fill in one box per answer.

Fill in as follows:

Date of birth: day/month/year

Weight (kg)

Height (cm)

Civil status: single, married, widowed, divorced, other

1. Since the last questionnaire, identify if you have been diagnosed for the first time by a physician with one of the following conditions or have you undergone one of the following circumstances:
   1. Motor vehicle accident with hospitalization of >24 hr
   2. Other motor vehicle accident without hospitalization but requiring leave of absence from work
   3. Sports injury requiring medical therapy
   4. Other accident with injury, including any fracture
   5. Hypertension: increase of > 8.5 diastolic or 13 systolic
   6. Osteoporosis
   7. Elevated total cholesterol (>200mg/dL)
   8. Myocardial infarction
   9. Angina pectoris
   10. Cardiopulmonary bypass surgery
   11. Atrial fibrillation
   12. Aortic aneurysm
   13. Heart failure
   14. Pulmonary embolism
   15. Deep vein thrombosis
   16. Diabetes (excluding gestational diabetes)
   17. Gestational diabetes
   18. Cerebral vascular accident (thrombosis-embolic-hemorrhagic)
   19. Intermittent claudication (peripheral vascular disease)
   20. Pregnancy (list the date of delivery or the expected due date)
   21. Consultation for fertility issues
   22. Cataract surgery
   23. Diagnosis of cataracts without surgery
   24. Increase of > ½ diopters of myopia
   25. Glaucoma
   26. Macular degeneration
   27. Chronic bronchitis or emphysema
   28. Asthma
   29. Gastric or duodenal ulcer
   30. Renal colic
   31. Gallstones
   32. Anorexia nervosa or bulimia
   33. Anxiety
   34. Depression
   35. Colorectal polyps
   36. Malignant tumor (specify the type)
   37. Other illness (please specify)

Thank you for your continuous participation!

C_12 questionnaire. Project SUN

Instructions:

Fill in the boxes ONLY with pencil.

Completely erase incorrect answers.

Leave in blank the box on the right upper quadrant of this sheet.

Do not fold this sheet.

Fill in the boxes correctly.

Fill in one box per answer.

Fill in as follows:

Date of birth: day/month/year

Weight (kg)

1. How often do you eat at fast food restaurants (pizzerias, burger joints)?
2. How often do you consume carbonated beverages (coolers, sodas, tonics, bitters…) excluding “light” beverages (1 can, 33 cl)?
3. How often do you consume “light” beverages (1can, 33cl)?
4. How often do you consume bottled juices?
5. Since the last questionnaire, identify if you have been diagnosed by a physician with one of the following diseases or conditions (specify approximate diagnosis date):
   1. Motor vehicle accident with hospitalization of >24 hr
   2. Other motor vehicle accident without hospitalization but requiring leave of absence from work
   3. Sports injury requiring medical therapy
   4. Other accident with injury, including any fracture
   5. Hypertension: increase of > 8.5 diastolic or 13 systolic
   6. Osteoporosis
   7. Elevated total cholesterol (>200mg/dL)
   8. Myocardial infarction
   9. Angina pectoris
   10. Cardiopulmonary bypass surgery
   11. Atrial fibrillation
   12. Aortic aneurysm
   13. Heart failure
   14. Pulmonary embolism
   15. Deep vein thrombosis
   16. Diabetes (excluding gestational diabetes)
   17. Gestational diabetes
   18. Cerebral vascular accident (thrombosis-embolic-hemorrhagic)
   19. Intermittent claudication (peripheral vascular disease)
   20. Pregnancy (list the date of delivery or the expected due date)
   21. Consultation for fertility issues
   22. Cataract surgery
   23. Diagnosis of cataracts without surgery
   24. Increase of > ½ diopters of myopia
   25. Glaucoma
   26. Macular degeneration
   27. Chronic bronchitis or emphysema
   28. Asthma
   29. Gastric or duodenal ulcer
   30. Renal colic
   31. Gallstones
   32. Anorexia nervosa or bulimia
   33. Anxiety
   34. Depression
   35. Colorectal polyps
   36. Malignant tumor (specify the type)
   37. Other illness (please specify)
6. Identify if you have been diagnosed by a physician with one of the following conditions or circumstances (specify approximate diagnosis date):
   1. Loss of memory
   2. Dementia
   3. Alzheimer’s disease
   4. Parkinson’s disease
   5. Rheumatologic disease
   6. Arthrosis
   7. Arthritis

Thank you for your continuous participation!

C_14 questionnaire. Project SUN

1. Date of birth
2. Weight (kg)
3. Civil status
   1. Single
   2. Married
   3. Widowed
   4. Divorced
   5. Other
4. Number of children
5. Have you smoked cigarettes in the last 4 weeks?
   1. No, I have never smoked
   2. No, I stopped smoking ….. years ago
   3. Yes, I have smoked for….
      1. Number of cigarettes
6. Please, mark only one option for each food
   1. Butter, margarine or cream (1 serving, 12g)
   2. Olive oil to cook, dress salads or to eat with bread (1 serving: 1 tablespoon)
   3. Homemade tomato sauce or stir-fry (garlic, onion) cooked with olive oil
   4. Vegetables (1 serving, 200g)
   5. Legumes (lentils, chickpeas, beans, etc; 1 serving, 60g)
   6. Sugar added to beverages (coffee, tea) or to food (yogurt): 1 teaspoon
   7. Garlic (individual piece)
   8. Parsley, oregano, etc (a pinch)
   9. Wheat rice (raw, 60g)
   10. Wheat pasta (raw, 90g)
   11. Wheat cereals: muesli, oatmeal (30g)
   12. Fruit (one)
   13. Nuts (1 serving, 30g)
   14. Red meats, sausages (1 serving, 100-150g)
   15. Chicken or turkey meat, rabbit (1 serving, 100-150g)
   16. Fish-seafood (1 serving of fish: 100-150g; 1 serving of seafood: 4-5 or 200g)
   17. Packaged food (1 serving)
   18. Packaged pastries, including cookies, cakes, flans and sweetened desserts
   19. Natural juice (1 glass)
   20. Carbonated beverages and/or sweetened (coolers, sodas, tonics, bitters) (1 can, 33cl)
   21. “Light beverages” (1 can, 33cl)
   22. Energetic beverages (Red Bull, Burn, etc)(1 can, 25cl)
   23. Beer (1 can, 33cl)
   24. Beer without alcohol (1 can, 33cl)
   25. Red wine (1 glass, 100ml)
   26. Other types of wine (1 glass, 100ml)
   27. Wine with meals (1 glass, 100ml)
   28. Distillated liquors: whisky, gin, cognac, licorice (1 cup, 50ml)
7. On average in a typical week, how often (days per week) do you drink alcohol (wine, beer or distillated liquors), including alcohol with meals?
   1. Never or almost never
   2. 1……
8. How often (days per week) do you drink wine with meals?
   1. Never or almost never
   2. 1….
9. What was the maximum number of alcoholic beverages (adding wine, beer and liquor) that you drank on a week day, weekend day or special event (festivity, wedding)
   1. None
   2. 1….
10. What percentage of olive oil that you use is “virgin extra”?
    1. I do not consume
    2. <25%....
11. Do you reuse the oil with which you fry foods?
    1. Never
    2. Sometimes
    3. Always
12. How often do you reuse the same oil?
    1. I do not reuse the oil
    2. 1-2
    3. 3-5
    4. > 6
13. Do you exercise?
    1. No
    2. Yes
       1. On average, how much time have you dedicated to the following activities in the past year?
          1. Walk or stroll outdoors (including golf)
          2. Run or jog slowly
          3. Run competitively or quickly (athletics, etc.)
          4. Ride a bicycle
          5. Ride a stationary bicycle
          6. Swim
          7. Tennis, racquet ball, squash, other racket or ball sports
          8. Soccer or futsal
          9. Other team sports (basketball, handball…)
          10. Ballet, dancing, aerobics
          11. Hiking
          12. Gymnastics
          13. Yard work or pool maintenance, home improvement
          14. Skiing, ice skating
          15. Judo, karate or other marshal arts
          16. Sailing
          17. Other activities not listed
14. Have you ever been diagnosed by a physician with gingivitis?
    1. No
    2. Yes
       1. Month and year
15. Have you noticed loss of memory recently?
    1. No
    2. Yes
       1. Month and year
16. Since the previous questionnaire, have you been diagnosed for the first time by a physician with one of the following conditions or have you undergone one of the following circumstances (remember that the date of your previous questionnaire that you sent to us is attached to your identification number in the upper right portion of the first page. Thus, the date that you list regarding the following questions, should be after the aforementioned date).
    1. Motor vehicle accident with hospitalization of >24 hr
    2. Other motor vehicle accident without hospitalization but requiring leave of absence from work
    3. Sports injury requiring medical therapy
    4. Other accident with injury, including any fracture
    5. Hypertension: increase of > 8.5 diastolic or 13 systolic
    6. Osteoporosis
    7. Elevated total cholesterol (>240mg/dL)
    8. Myocardial infarction
    9. Angina pectoris
    10. Cardiopulmonary bypass surgery
    11. Atrial fibrillation
    12. Aortic aneurysm
    13. Heart failure
    14. Pulmonary embolism
    15. Deep vein thrombosis
    16. Diabetes (excluding gestational diabetes)
    17. Gestational diabetes
    18. Cerebral vascular accident (thrombosis-embolic-hemorrhagic)
    19. Intermittent claudication (peripheral vascular disease)
    20. Pregnancy (list the date of delivery or the expected due date)
    21. Consultation for fertility issues
    22. Cataract surgery
    23. Diagnosis of cataracts without surgery
    24. Increase of > ½ diopters of myopia
    25. Glaucoma
    26. Macular degeneration
    27. Chronic bronchitis or emphysema
    28. Asthma
    29. Gastric or duodenal ulcer
    30. Renal colic
    31. Gallstones
    32. Anorexia nervosa or bulimia
    33. Anxiety
    34. Depression
    35. Colorectal polyps
    36. Loss of memory
    37. Dementia
    38. Alzheimer’s disease
    39. Parkinson’s disease
    40. Rheumatologic disease
    41. Arthrosis
    42. Arthritis
    43. Rheumatoid arthritis
    44. Malignant tumor (specify the type)
    45. Other illness (please specify)
17. What medications or dietary supplements (including vitamins, fibers, wheat bran, etc) do you consume on a regular basis?
    1. I do not frequently consume medications or supplements
    2. I consume the following medications or supplements daily (if you need more space, please add a separate sheet)

Thank you for your collaboration!

C_16 questionnaire. Project SUN

1. ID
2. Date of birth
3. Weight (kg)
4. Minimum weight in the last 2 years (kg)
5. Maximum weight in the last 2 years (kg)
6. Have you lost weight intentionally
   1. Yes (specify how much and how many times)
   2. No (proceed to question #8)
7. What did you do to lose weight? (you can choose many options)
   1. I have not lost weight intentionally
   2. Intentional weight loss (disease, stress, depression)
   3. Hypocaloric diet
   4. Increase in exercise
   5. Bariatric surgery/gastric bypass
   6. Gastric balloon
   7. Fast
   8. Medication
   9. Other
8. When you were born, were you breastfed?
   1. No
   2. Yes
      1. Months
9. Do you know how much you weighted when you were born? (in grams)
10. Have you smoked any cigarettes in the last 4 weeks?
    1. No, I have never smoked
    2. No, I stopped smoking …. years ago
    3. Yes, I have smoked since…
       1. Number of cigarettes
11. What is the highest degree of education you have completed?
    1. I have not finished any degree (proceed to question # 13)
    2. Doctorate
    3. Licensure – Superior School
    4. Diploma (engineering, university colleges)
12. Have you finished any of the following studies?
    1. Medicine
    2. Pharmacology
    3. Biology
    4. Nursing
    5. Nutrition-dietitian
    6. Psycology
    7. Other biomedical degrees
    8. No biomedical degrees
13. How many hours on average do you sleep on a regular weekday?
    1. Sleep at night (hr)
    2. Nap (hr)
14. How many hours on average do you sleep on a regular weekend day?
    1. Sleep at night (hr)
    2. Nap (hr)
15. How easy do you fall asleep?
    1. Time needed in minutes
    2. Time needed to go back to sleep at night in minutes
16. Do you work on shifts?
    1. No
    2. Yes
       1. AM
       2. PM
       3. Evenings
       4. Rotating
       5. Weekends
       6. Other
17. What kind of media do you use?
    1. Facebook
    2. Twitter
    3. ResearchGate
    4. Academia
    5. Linkedin
    6. Other
    7. None
18. How much time do you spend on media (hr per week)?

Below, you will find statements that you could apply to yourself. You must describe yourself as accurate as possible using one of the four options given (0=total disagreement, 1=moderate disagreement, 2=moderate agreement, 3=total agreement).

1. People would describe me as inpatient
2. I think I act impulsively
3. Even though I could do better, I can’t help making rush decision
4. I often feel that nothing that I do really matters
5. Others view me as irresponsible
6. I am not very organized in advance
7. Frequently, my thoughts do not make sense to others
8. I worry just about everything
9. I become emotional easily, even over small matters
10. Being alone frightens me more than anything
11. I get stuck doing things in a particular manner, even if it’s clear it is not functioning
12. I have seen things that are not really there
13. I stay away from sentimental relationships
14. I am not interested in making friends
15. I easily become irritable with all type of things
16. I do not like becoming intimate with others
17. It does not matter if I hurt others feelings
18. Rarely do I become excited over nothing
19. I often try to attract attention
20. Very often I try to relate to people who are less important than I
21. I often have thoughts that make sense to me but that others find strange
22. I use people to get what I want
23. I often day dream and when I react I notice that much time has passed
24. The things that surround me often seem unreal or more real than normal
25. I find it easy to take advantage of others
26. Since the previous questionnaire, have you been diagnosed for the first time by a physician with one of the following conditions or have you undergone one of the following circumstances (remember that the date of your previous questionnaire that you sent to us is attached to your identification number in the upper right portion of the first page. Thus, the date that you list regarding the following questions, should be after the aforementioned date).
    1. Motor vehicle accident with hospitalization of >24 hr
    2. Other motor vehicle accident without hospitalization but requiring leave of absence from work
    3. Sports injury requiring medical therapy
    4. Other accident with injury, including any fracture
    5. Hypertension: increase of > 8.5 diastolic or 13 systolic
    6. Osteoporosis
    7. Elevated total cholesterol (>240mg/dL)
    8. Myocardial infarction
    9. Angina pectoris
    10. Cardiopulmonary bypass surgery
    11. Atrial fibrillation
    12. Aortic aneurysm
    13. Heart failure
    14. Pulmonary embolism
    15. Deep vein thrombosis
    16. Diabetes (excluding gestational diabetes)
    17. Gestational diabetes
    18. Cerebral vascular accident (thrombosis-embolic-hemorrhagic)
    19. Intermittent claudication (peripheral vascular disease)
    20. Pregnancy (list the date of delivery or the expected due date)
    21. Consultation for fertility issues
    22. Cataract surgery
    23. Diagnosis of cataracts without surgery
    24. Increase of > ½ diopters of myopia
    25. Glaucoma
    26. Macular degeneration
    27. Chronic bronchitis or emphysema
    28. Asthma
    29. Gastric or duodenal ulcer
    30. Renal colic
    31. Gallstones
    32. Anorexia nervosa or bulimia
    33. Anxiety
    34. Depression
    35. Colorectal polyps
    36. Loss of memory
    37. Dementia
    38. Alzheimer’s disease
    39. Parkinson’s disease
    40. Rheumatologic disease
    41. Arthrosis
    42. Arthritis
    43. Rheumatoid arthritis
    44. Malignant tumor (specify the type)
    45. Other illness (please specify)
27. Have you been diagnosed with migraine headaches?
    1. No
    2. Yes
       1. Month, year

Women should only answer the following questions.

1. Have you undergone menopause (loss of menstrual periods)?
   1. No
   2. Yes
      1. Month, year
2. What medications or dietary supplements (including vitamins, fibers, wheat bran, etc) do you consume on a regular basis?
   1. I do not frequently consume medications or supplements
   2. I consume the following medications or supplements daily (if you need more space, please add a separate sheet)
3. Nutrition (please, mark only one option for each food). For each one, mark the box which indicates the mean frequency of consumption during the last year. Assume the size of a regular serving.
   1. Wheat bread (3 slices, 60g)
   2. Wheat rice (serving of 60g)
   3. Wheat pasta (serving of 60g)
   4. Wheat cereal (muesli, oatmeal) (30g)
   5. Functional foods (enriched milk, margarine with phytosterols, enriched cereals)
   6. Carbonated beverages and/or sweetened (coolers, sodas, tonics, bitter) excluding “light” beverages (1 can, 33cl)
   7. Light beverages (1 can, 33cl)
   8. Fruit (one serving)
   9. Vegetables (1 serving: 200g)
   10. How often do you eat at fast food restaurants (pizzerias, burger joints)?
   11. Sugar added to food or beverages (1 teaspoon)
   12. Non-caloric sweeteners
   13. Salt in meals (after cooking)

**SUN FOOD FREQUENCY QUESTIONNAIRE -Translation**

Please, mark only one option for each food. *For each food, mark the box that indicates the average consumption during the past year. We try take into account variation between summer/winter. For example, if you eat ice cream 4 times a week during the three months of summer, the average usage would be once a week per year.*

1. Whole milk (one cup, 200ml)
2. Half milk (one cup, 200ml)
3. Skim milk (one cup, 200ml)
4. Condensed milk (1 tablespoon)
5. Whipped cream or creamer (1/2 cup)
6. Milk shakes (one cup, 200ml)
7. Yogurt (1, 125g)
8. Low fat yogurt (1, 125g)
9. Petit suisse (1, 100g)
10. Curd or cottage cheese (half a cup)
11. Cream cheese or cheese slices (1 portion)
12. Other cheeses: cured or semi-cured (Manchego, Swiss cheese, etc) (50g)
13. White or fresh cheese (Burgos, goat cheese) (50g)
14. Custard, flan, pudding (1 cup, 200ml)
15. Ice cream (one)

Please, mark only one option for each food. *One plate or portion of a 100-250g, except when otherwise indicated.*

1. Chicken eggs (one)
2. Chicken or turkey WITH skin
3. SKINLESS chicken or turkey
4. Calf or cow meat
5. Pork meat
6. Lamb meat
7. Rabbit or hare
8. Liver
9. Other organs: brain, heart, etc.
10. Serrano ham
11. Baked ham (one slice)
12. Sausages (chorizo, salchichón, mortadela) (50g)
13. Sausages (50g)
14. Paté, foie-gras (25g)
15. Blood sausage (50g)
16. Hamburger (one)
17. Sobressada (50g), meatball (three)
18. Bacon, pancetta (50g)
19. White fish: whiting, hake, sea bream, grouper, flounder (1 plate, piece or portion)
20. Blue fish: sardines, tuna, mackerel, salmon (1 plate, piece or portion)
21. Cod
22. Salted or smoked fish: herring, salmon
23. Oysters, clams, mussels, etc (six)
24. Shrimp, lobster, crayfish
25. Octopus, calamari, squid, cuttlefish

*One plate or portion of 250g, except when otherwise indicated.*

1. Chard, spinach
2. Cauliflower, Brussel sprouts, broccoli
3. Lettuce, escarole, endive
4. Row tomato (one, 150g)
5. Carrots, pumpkin
6. Green beans
7. Eggplant, squash, cucumber
8. Peppers
9. Asparagus
10. Gazpacho
11. Other greens (borage, thistle)
12. French fries, home maid, bag (1 portion, 150g)
13. Baked or steamed potatoes (1 portion, 150g)

Please, mark only one option for each food. *1 plate or portion.*

1. Orange, grapefruit (one), or mandarin (two)
2. Banana
3. Apple, pear
4. Strawberries (six, fruit salad)
5. Peach, apricot, nectarine
6. Cherries, plums (1 plate)
7. Figs
8. Watermelon (1 slice, 200-250g)
9. Melon (1 slice, 200-250g)
10. Canned fruits (2 cans)
11. Dates, dry figs, raisins (150g)
12. Almonds, peanuts, hazelnuts, walnuts (50g)
13. Olives (ten)
14. Avocado
15. Mangos, papaya
16. Kiwis

How many days a week you do it fruit as dessert?

Please, mark only one option for each food. *1 plate or portion of 60g dry weight.*

1. Lentils
2. Chickpeas
3. Beans (pinto, white, black)
4. Peas
5. White bread (3 slices, 60g)
6. Wheat bread (3 slices, 60g)
7. Cereals (30g)
8. White rice (60g)
9. Pasta, noodles, macaroni, spaghetti (60g)
10. Pizza (1 slice, 200g)

Please, mark only one option for each food. 1 tablespoon or individual portion to deep, or dress salads, total:

1. Butter
2. Margarine
3. Olive oil
4. Sunflower oil
5. Corn oil
6. Lard
7. Other:

How often do you consume: fried foods at home, fried food at restaurants

At home, when frying, I use: olive oil, sunflower oil, corn oil, butter, margarine, others

Brand of olive oil that you usually use at home

Please, mark only one option for each food

1. Maria cookies (4-6 cookies, 50g)
2. Cookies with chocolate (4-6 cookies, 50g)
3. Packaged muffins (1-2 muffins)
4. Donuts (one)
5. Roll, croissant or other packaged pastries (one, 50g)
6. Home maid pastries or dessert
7. Cakes (one, 50g)
8. Churros (one portion, 100g)
9. Chocolates and truffles (30g)
10. Turron (1/8 bar)
11. Tea cookies, ice cream, mazapan (1 portion, 90g)

Please, mark only one option for each food

1. Glass of red wine
2. Glass of other type of wine
3. Glass of wine with meals
4. Beer (1 pitcher, 330ml)
5. Liquors: whisky, gin, cognac, licorice (1 glass, 50ml)
6. Carbonated beverages with sugar: coca cola, fanta (1 bottle, 200ml)
7. As above, but low calorie, diet beverages (1 bottle, 200ml)
8. Natural orange juice (1 glass, 200ml)
9. Other natural fruit juices (1 glass, 200 ml)
10. Bottled or canned fruit or vegetable juice (200ml)
11. Decaffeinated coffee (1 cup, 50ml)
12. Regular coffee (1 cup, 50ml)
13. Tap water (1glass, 200ml)
14. Bottled water (1 glass, 200ml)

List which brand of water bottle you usually drink.

Please, mark only one option for each food

1. Croquetas, bunuelas, empanadas
2. Soups and creams
3. Tomato sauce, ketchup (1 teaspoon)
4. Mayonnaise (1 teaspoon)
5. Spice: tabasco, red pepper
6. Salt (one pinch)
7. Sugar (one teaspoon)
8. Splenda
9. Jams (1 teaspoon)
10. Honey
11. Other foods frequently consumed

How frequently do you eat out?

Have you consumed vitamins and/or minerals (including calcium) regularly over the past year?

If yes, please list the brand

Normally, what do you do with the fat of cooked meat?

1. I eat it
2. I remove it

Do you try to consume much fiber?

Do you try to consume much fruit?

Do you try to consume much vegetables?

Do you try to consume much fish?

Do you usually eat snacks in between meals?

Do you follow a special diet? If yes, indicate the type of diet.

Do you avoid the use of butter?

Do you try to reduce your consumption of fat?

Do you try to reduce your consumption of meat?

Do you restrict salt in your diet?

Do you add sugar to some beverages?

Do you try to reduce consumption of sweets?
